# Supplementary material for: Genetic and pharmacological reduction of CDK14 mitigates synucleinopathy
Source: Cell Death Dis. 2024 Apr 4;15(4):246. doi: 10.1038/s41419-024-06534-8 (PMC10994937; doi:10.1038/s41419-024-06534-8)

Fig2

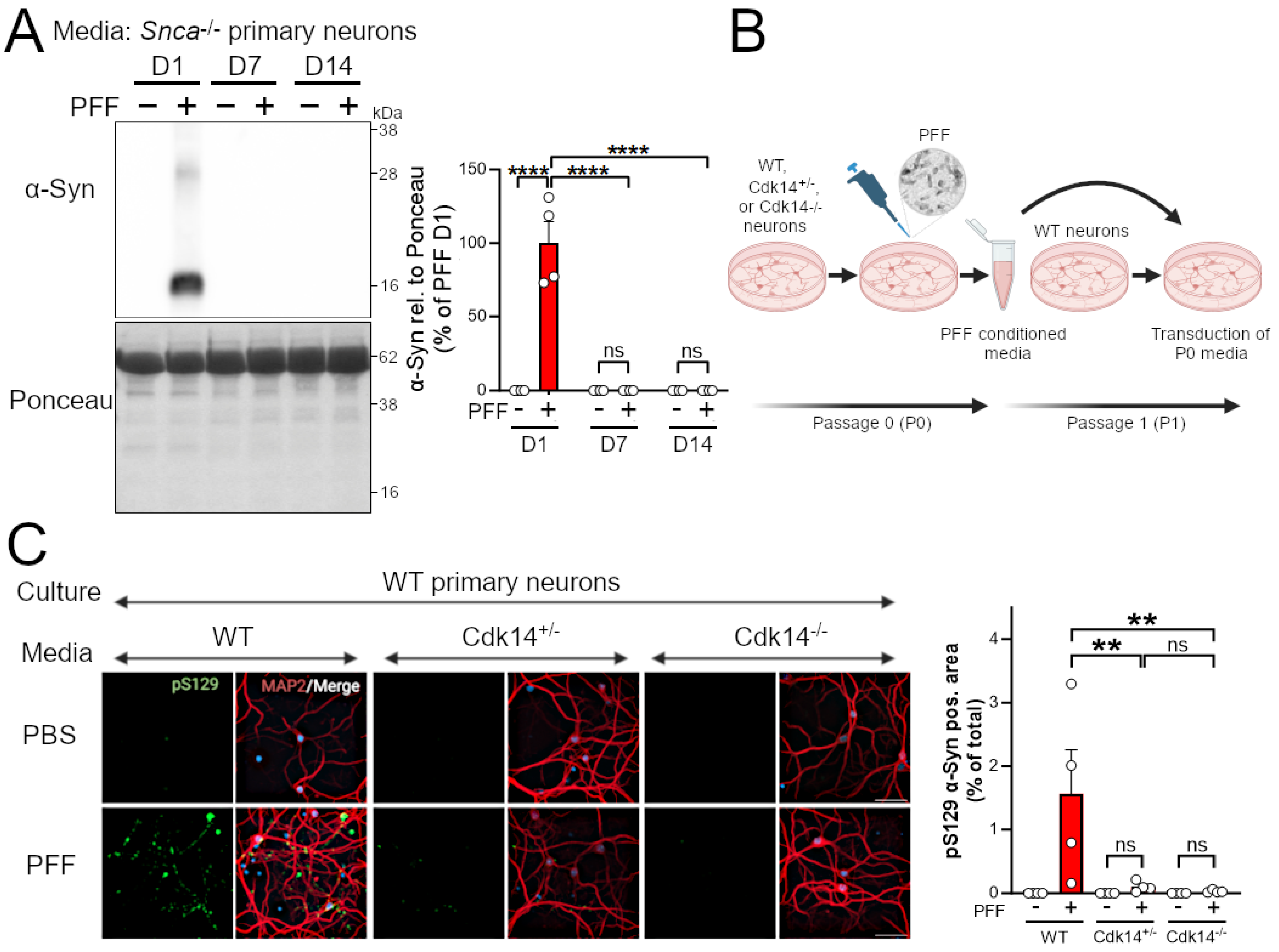
A
Media: Snca-/- primary neurons
D1 D7 D14
PFF − + − + − +
kDa
38
28
16
62
38
16
α-Syn
Ponceau
α-Syn rel. to Ponceau (% of PFF D1)
150
100
50
0
****
****
****
ns
ns
PFF - + - + - +
D1 D7 D14
B
PFF
WT, Cdk14+/-, or Cdk14-/- neurons
WT neurons
PFF conditioned media
Transduction of P0 media
Passage 0 (P0)
Passage 1 (P1)
C
Culture
WT primary neurons
Media
WT
Cdk14+/-
Cdk14-/-
pS129
MAP2/Merge
PBS
PFF
pS129 α-Syn pos. area (% of total)
4
3
2
1
0
**
**
ns
ns
ns
PFF - + - + - +
WT Cdk14+/- Cdk14-/-

Fig2A

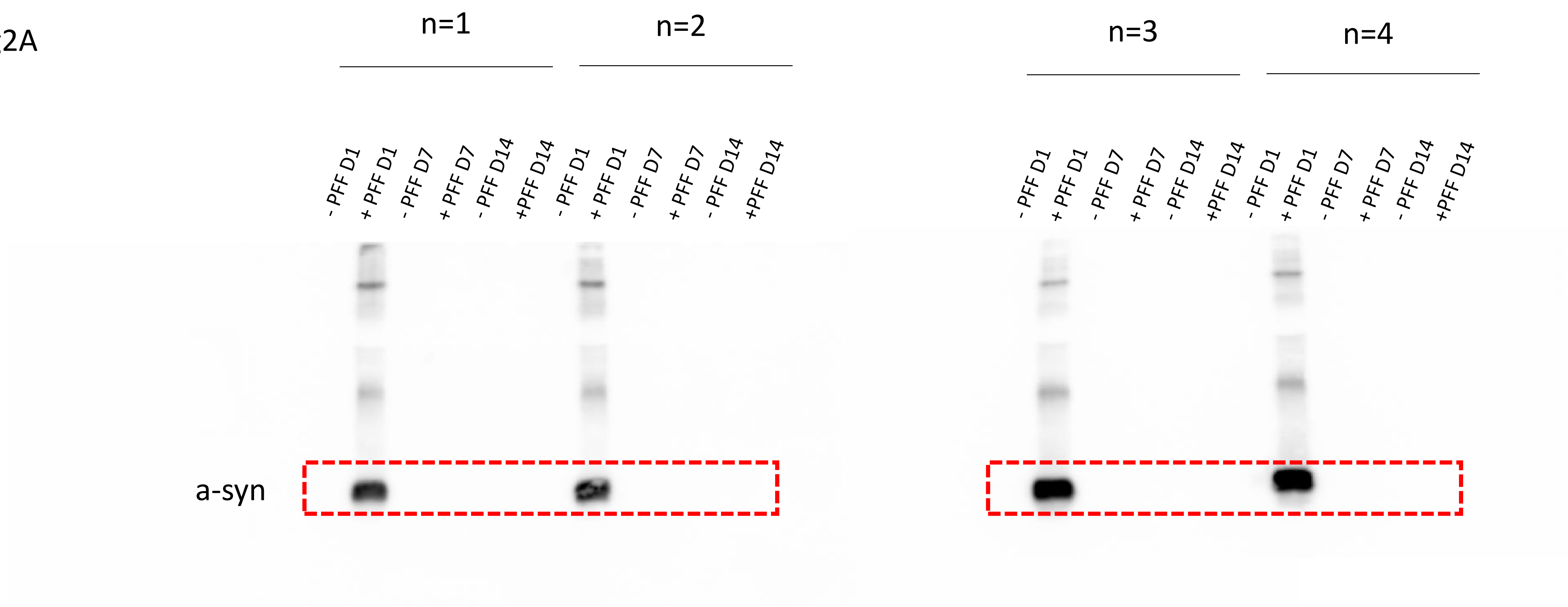
n=1
n=2
n=3
n=4
- PFF D1
+ PFF D1
- PFF D7
+ PFF D7
- PFF D14
+PFF D14
- PFF D1
+ PFF D1
- PFF D7
+ PFF D7
- PFF D14
+PFF D14
- PFF D1
+ PFF D1
- PFF D7
+ PFF D7
- PFF D14
+PFF D14
- PFF D1
+ PFF D1
- PFF D7
+ PFF D7
- PFF D14
+PFF D14
a-syn

Fig2A

Ponceau

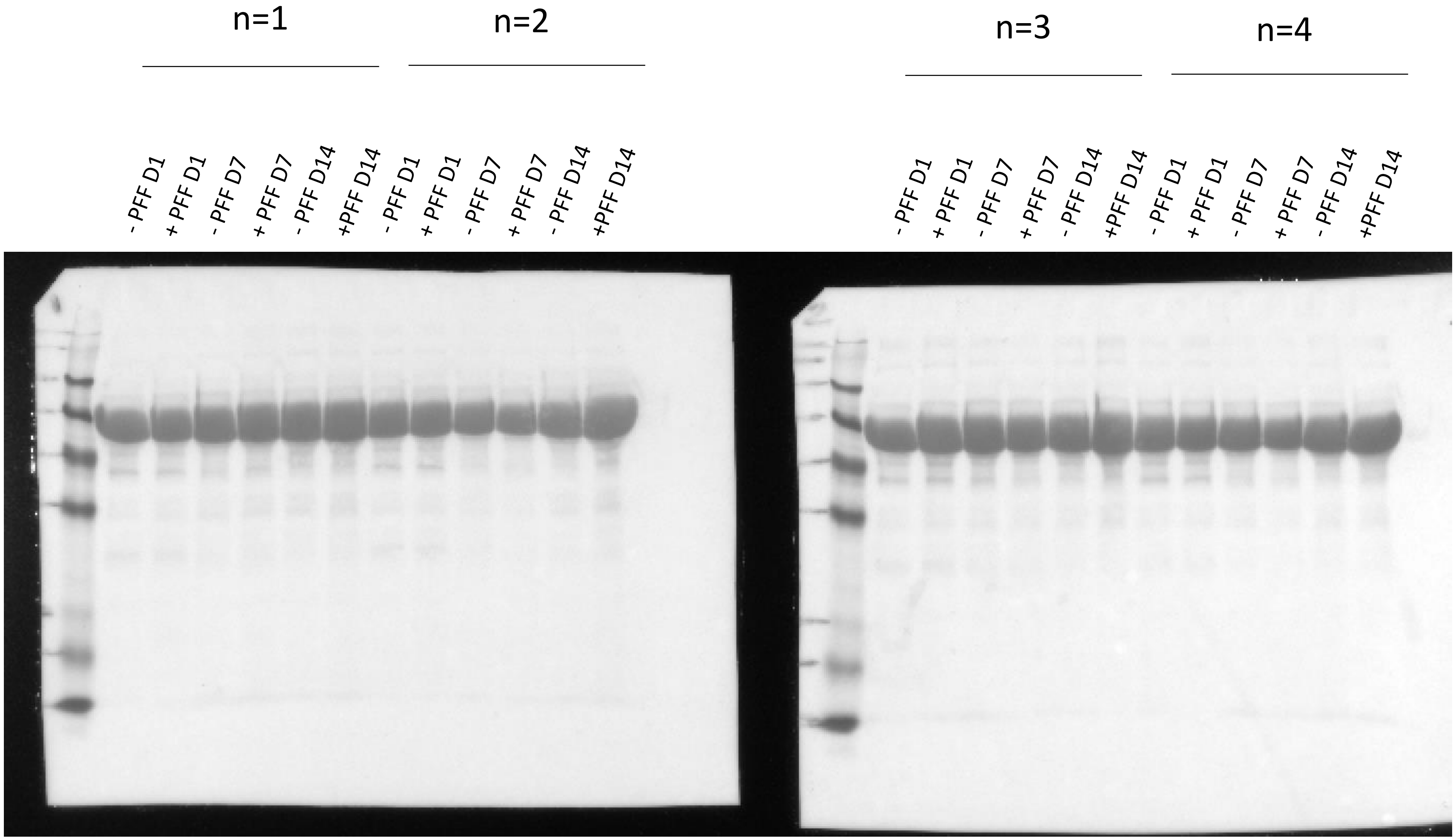

Fig3

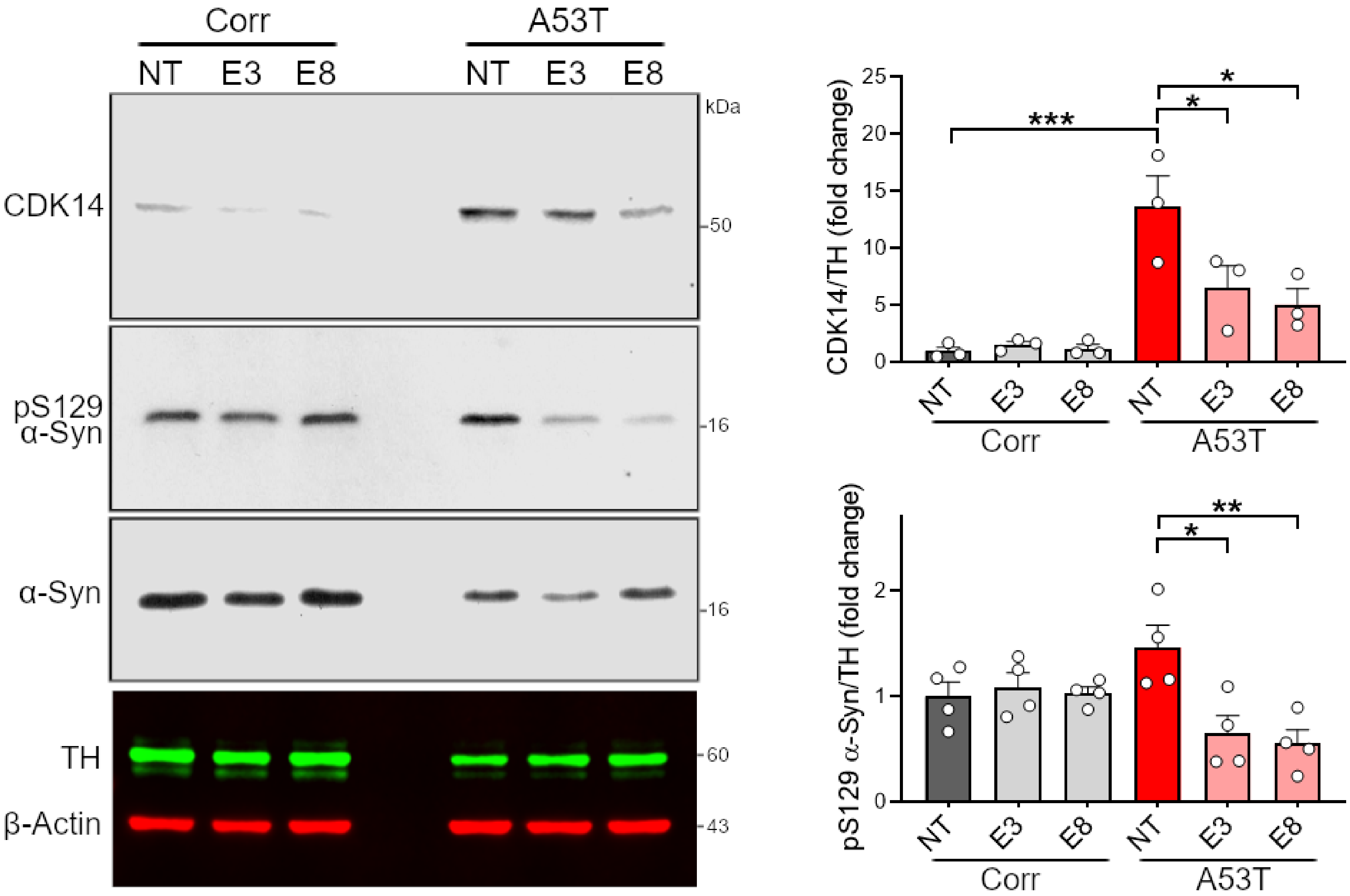
Corr
A53T
NT
E3
E8
NT
E3
E8
kDa
CDK14
-50
pS129
α-Syn
-16
α-Syn
-16
TH
-60
β-Actin
-43
CDK14/TH (fold change)
25
20
15
10
5
0
***
*
*
NT
E3
E8
NT
E3
E8
Corr
A53T
pS129 α-Syn/TH (fold change)
2
1
0
*
**
NT
E3
E8
NT
E3
E8
Corr
A53T

Fig3

CDK14 n=1

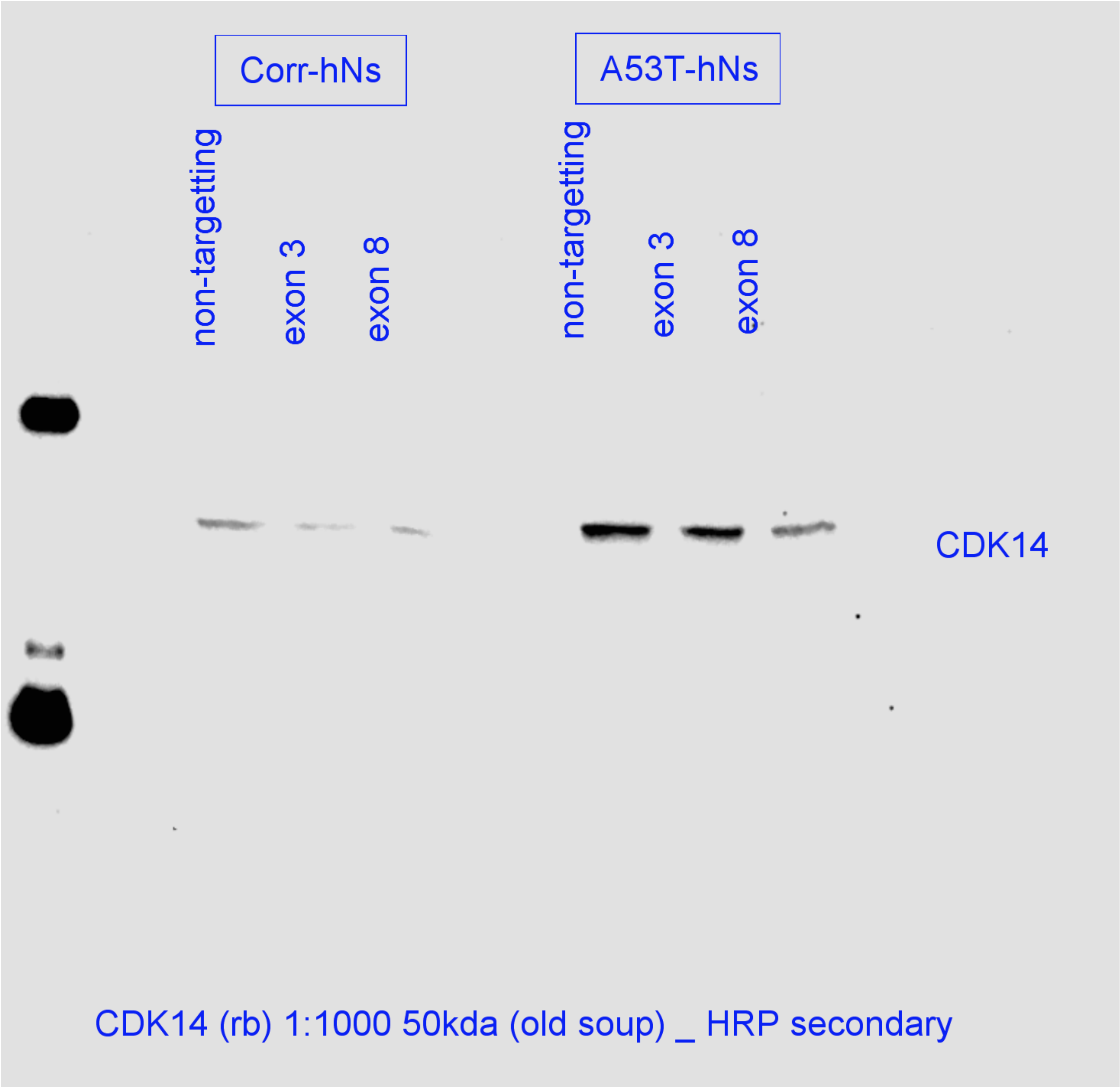

Fig3

pS129 a-syn n=1

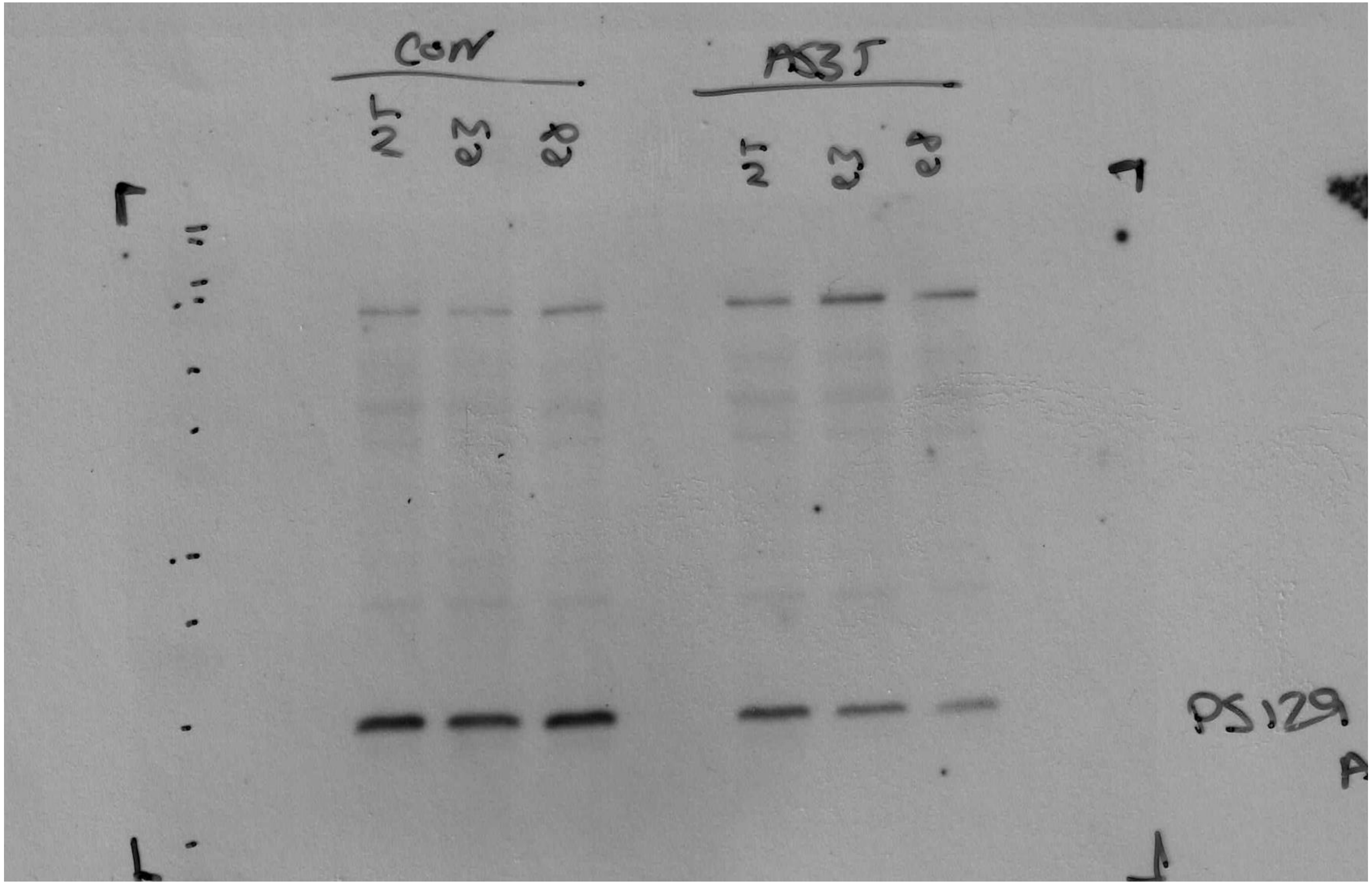

Fig3

a-syn n=1

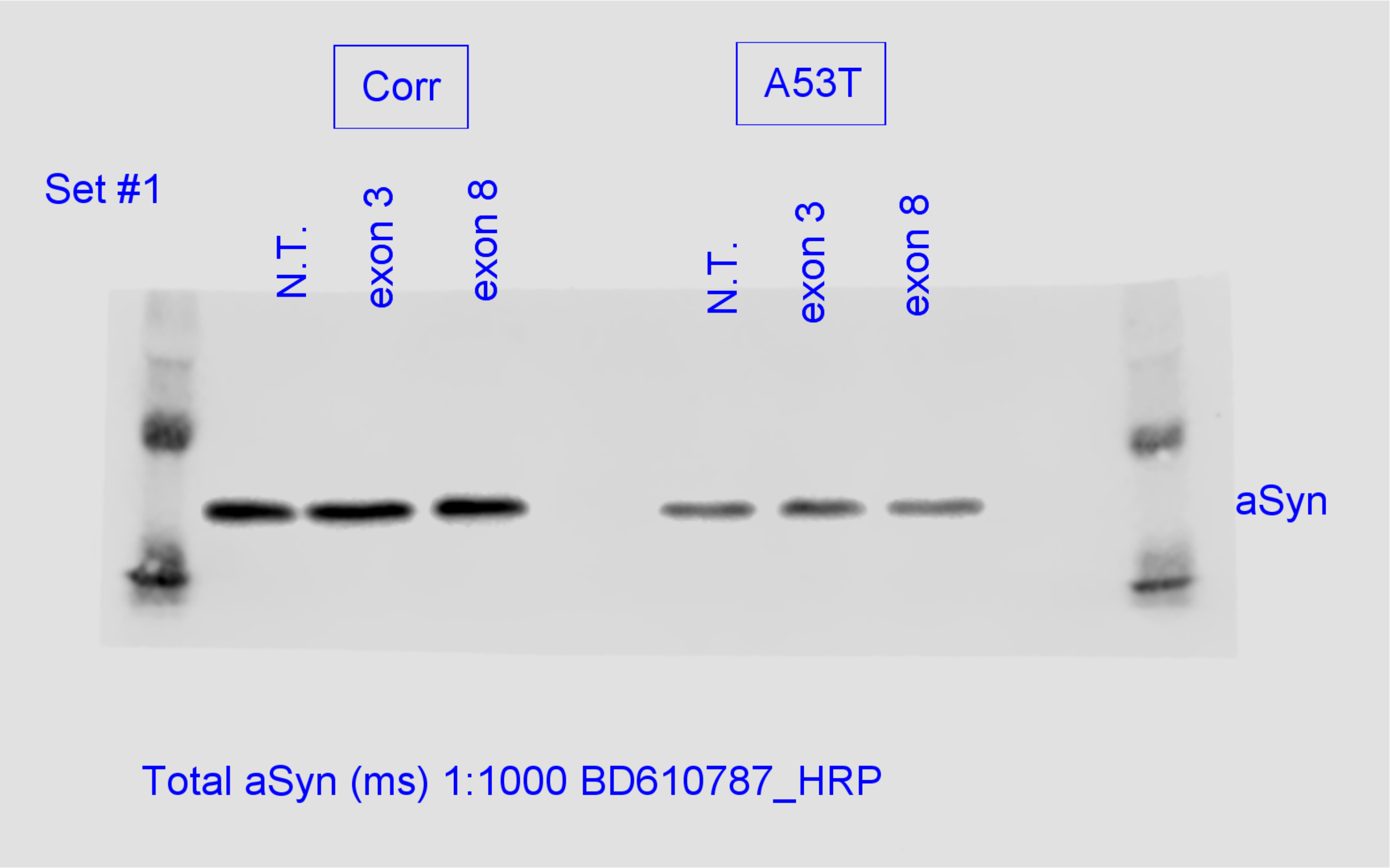
Corr
A53T
Set #1
N.T.
exon 3
exon 8
N.T.
exon 3
exon 8
aSyn
Total aSyn (ms) 1:1000 BD610787_HRP

Fig3

TH, beta-actin n=1

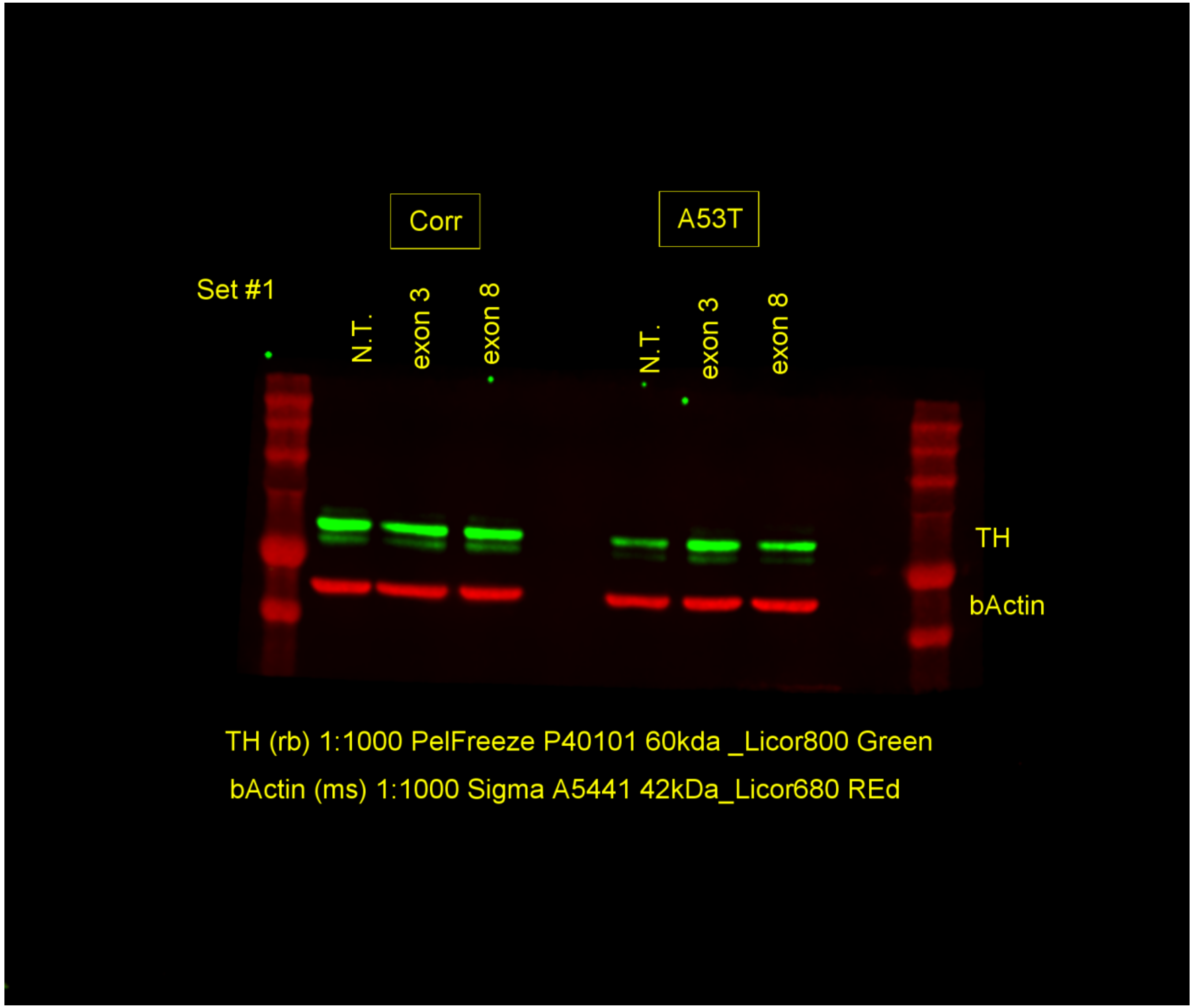

Fig3

CDK14 n=2

Higher exposure

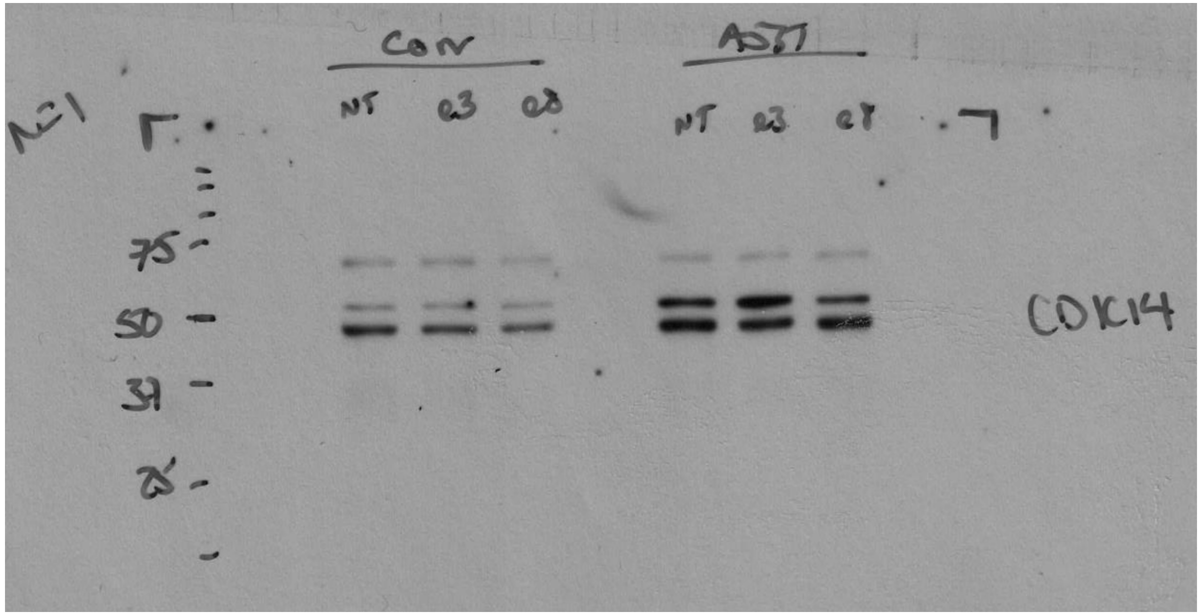

Lower exposure

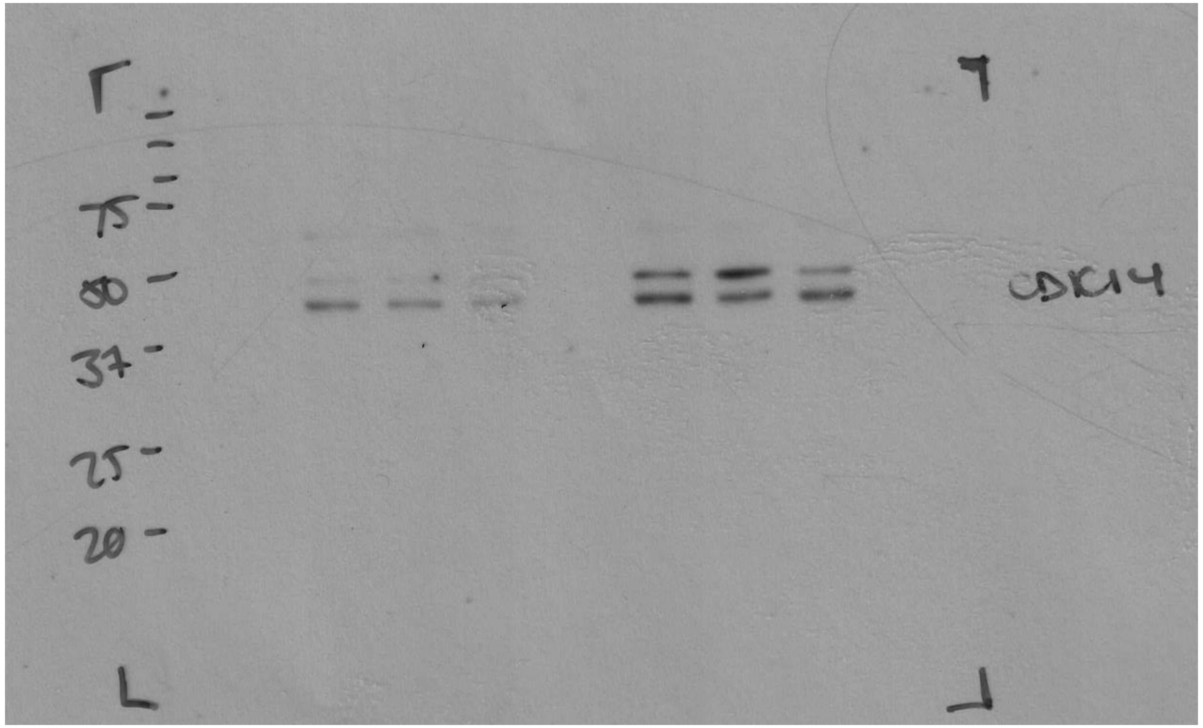

Fig3

pS129 a-syn n=2

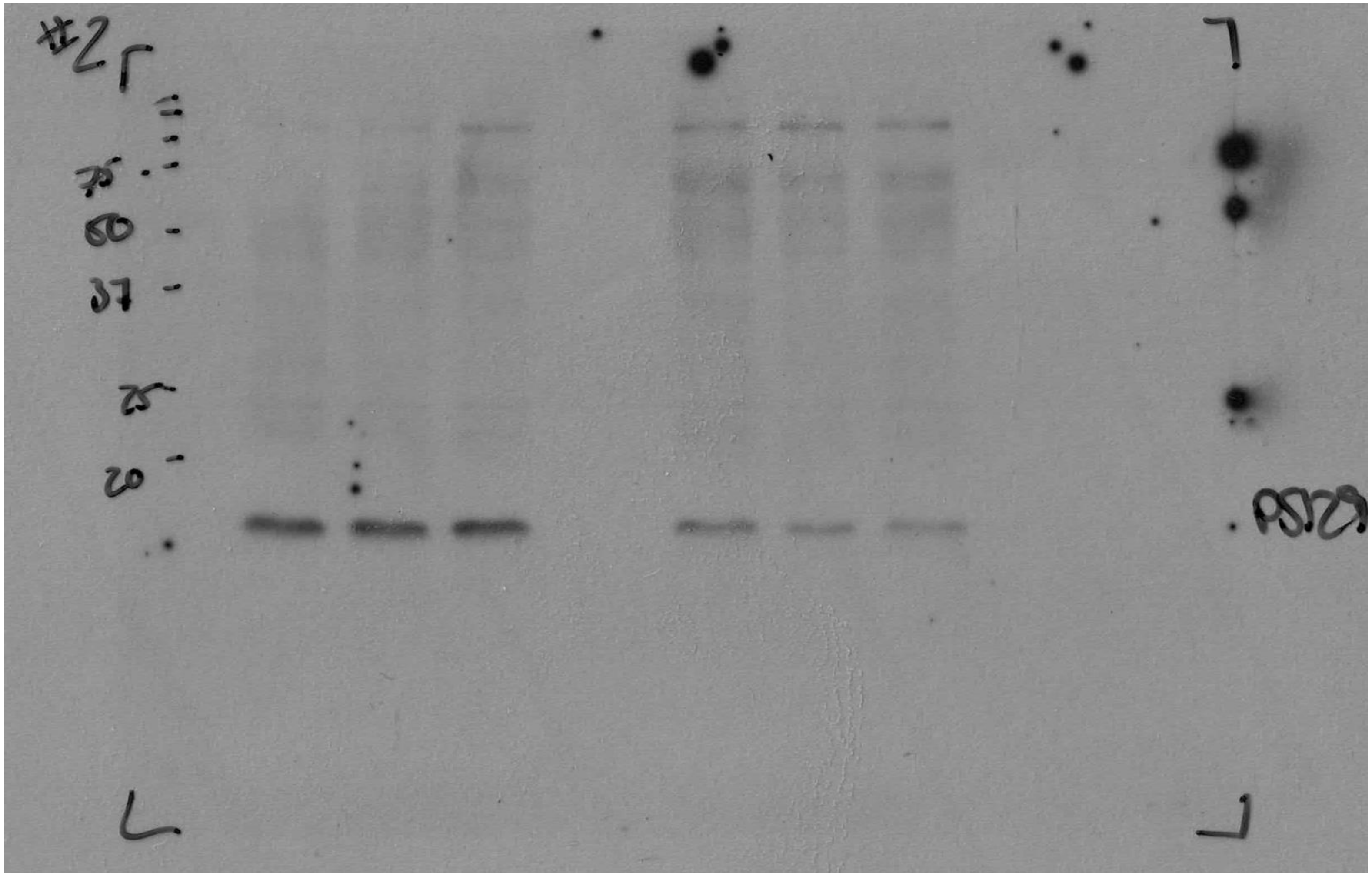

Fig3

a-syn n=2

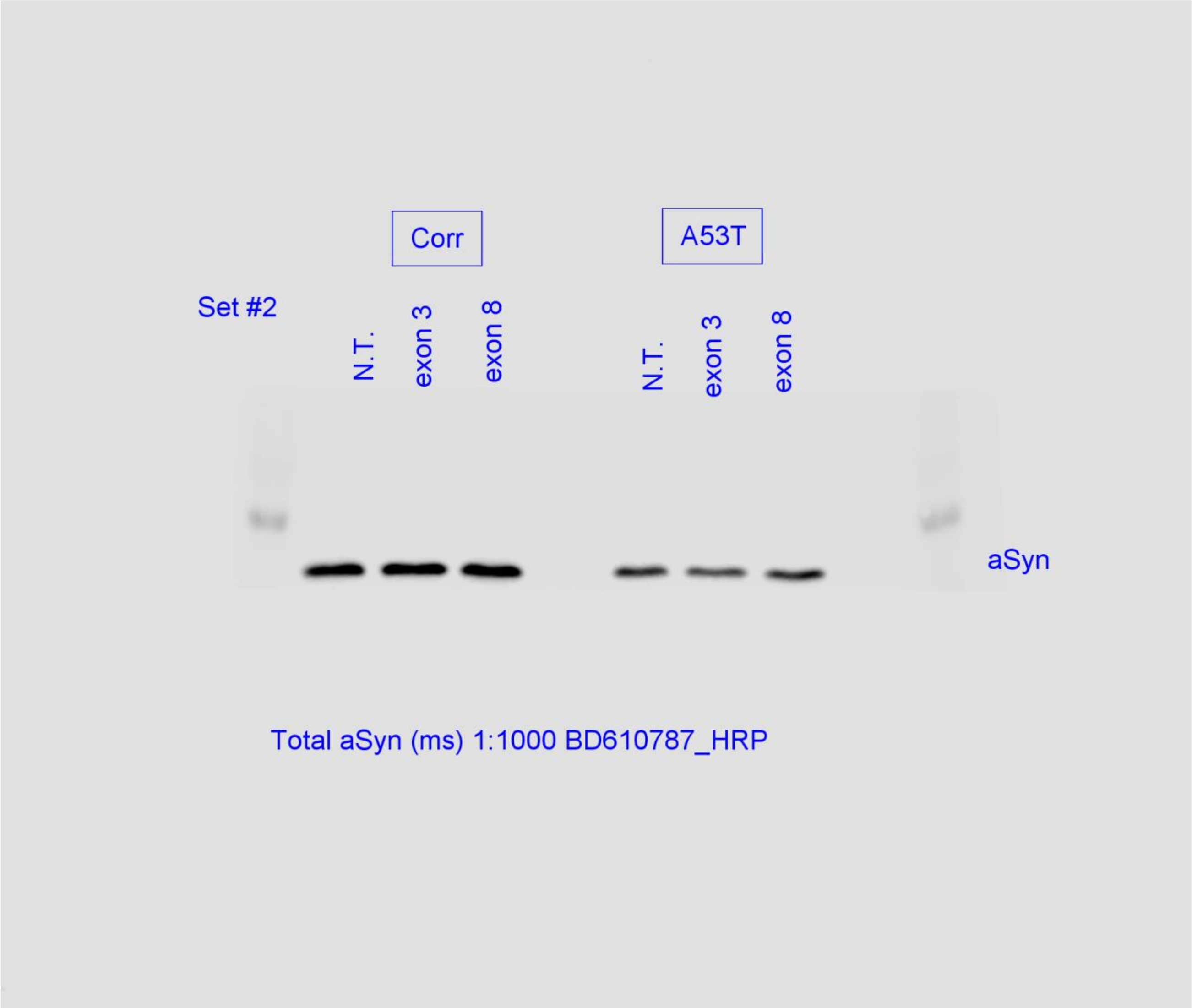
Corr
A53T
Set #2
N.T.
exon 3
exon 8
N.T.
exon 3
exon 8
aSyn
Total aSyn (ms) 1:1000 BD610787_HRP

Fig3

TH, beta-actin n=2

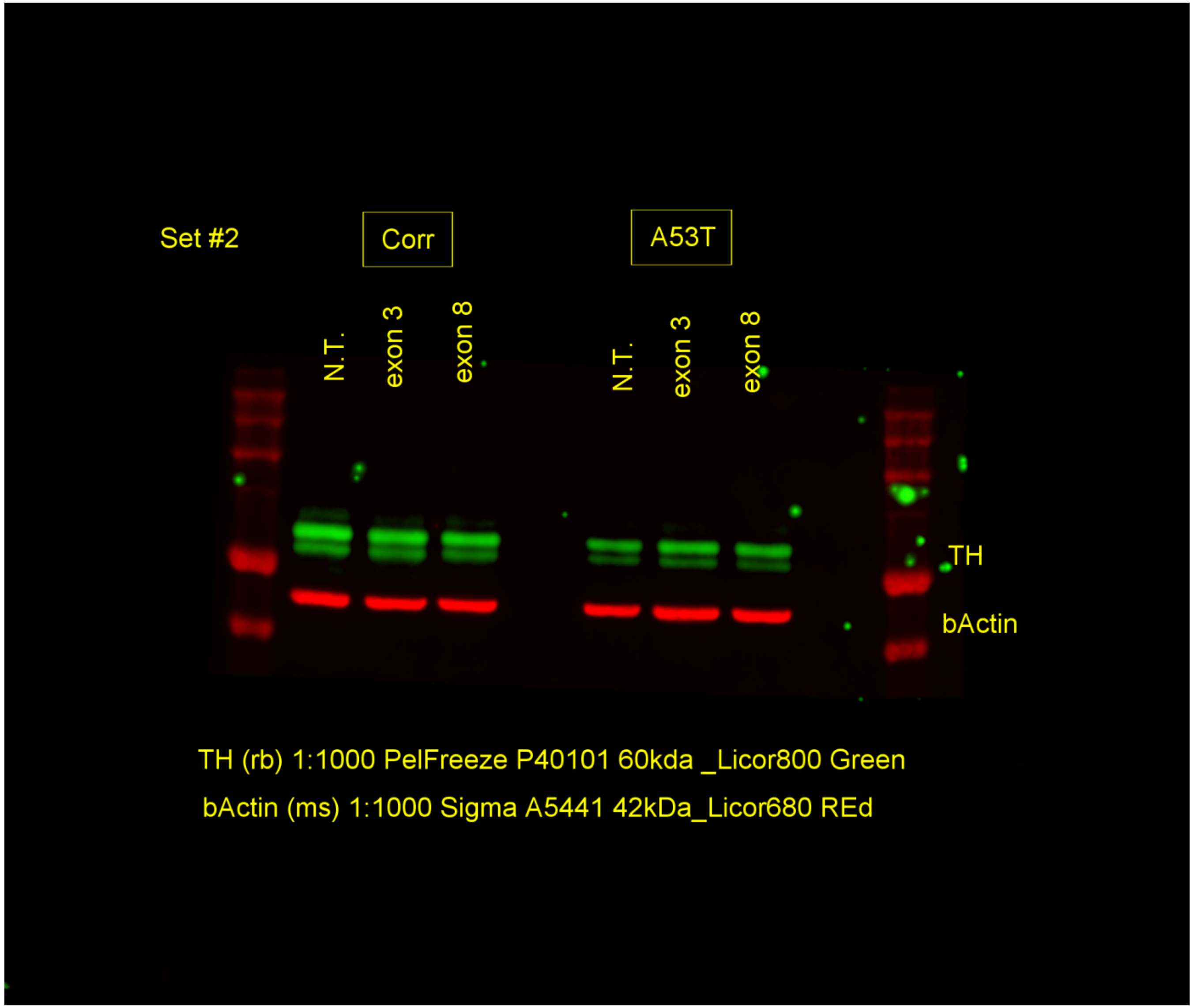

Fig3

CDK14 n=3

Higher exposure

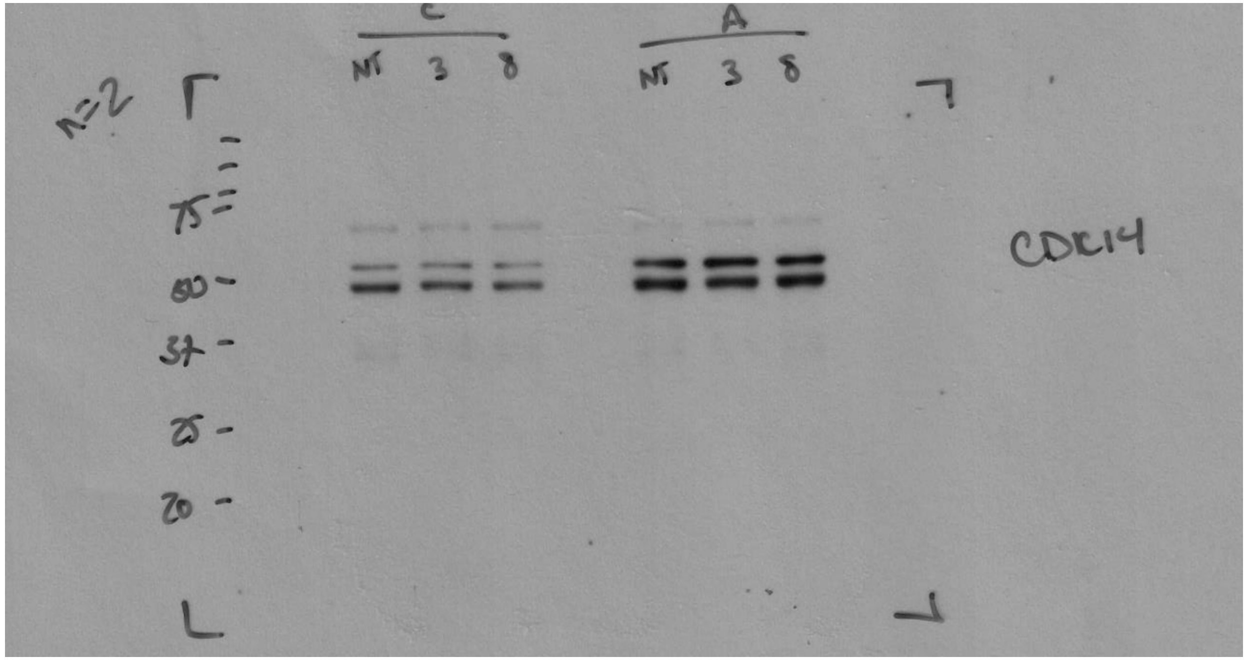

Lower exposure

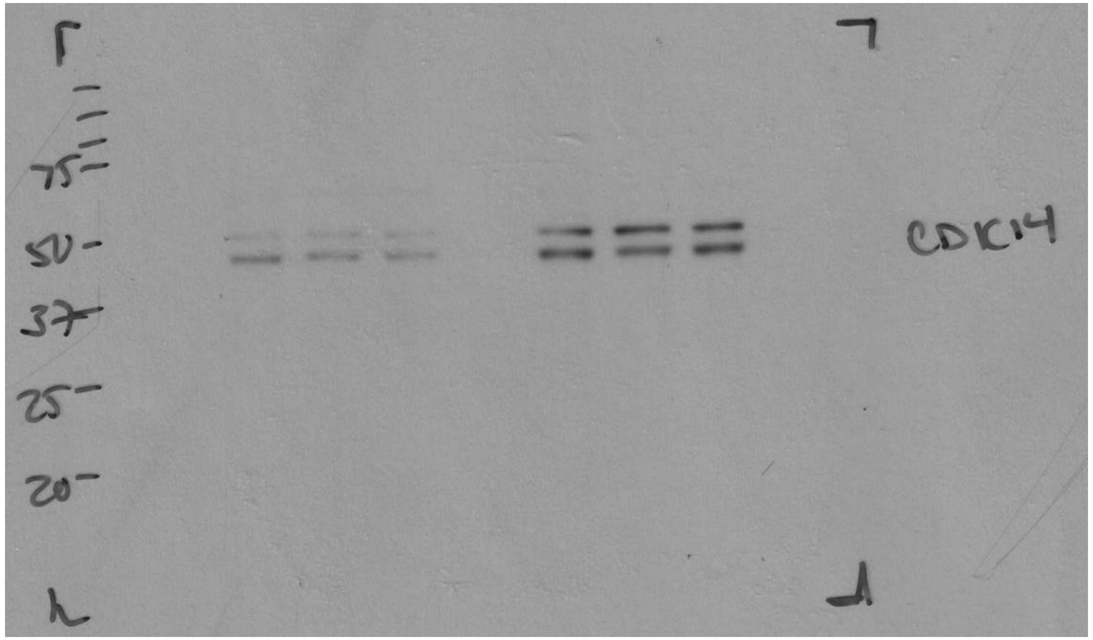

Fig3

pS129 a-syn n=3

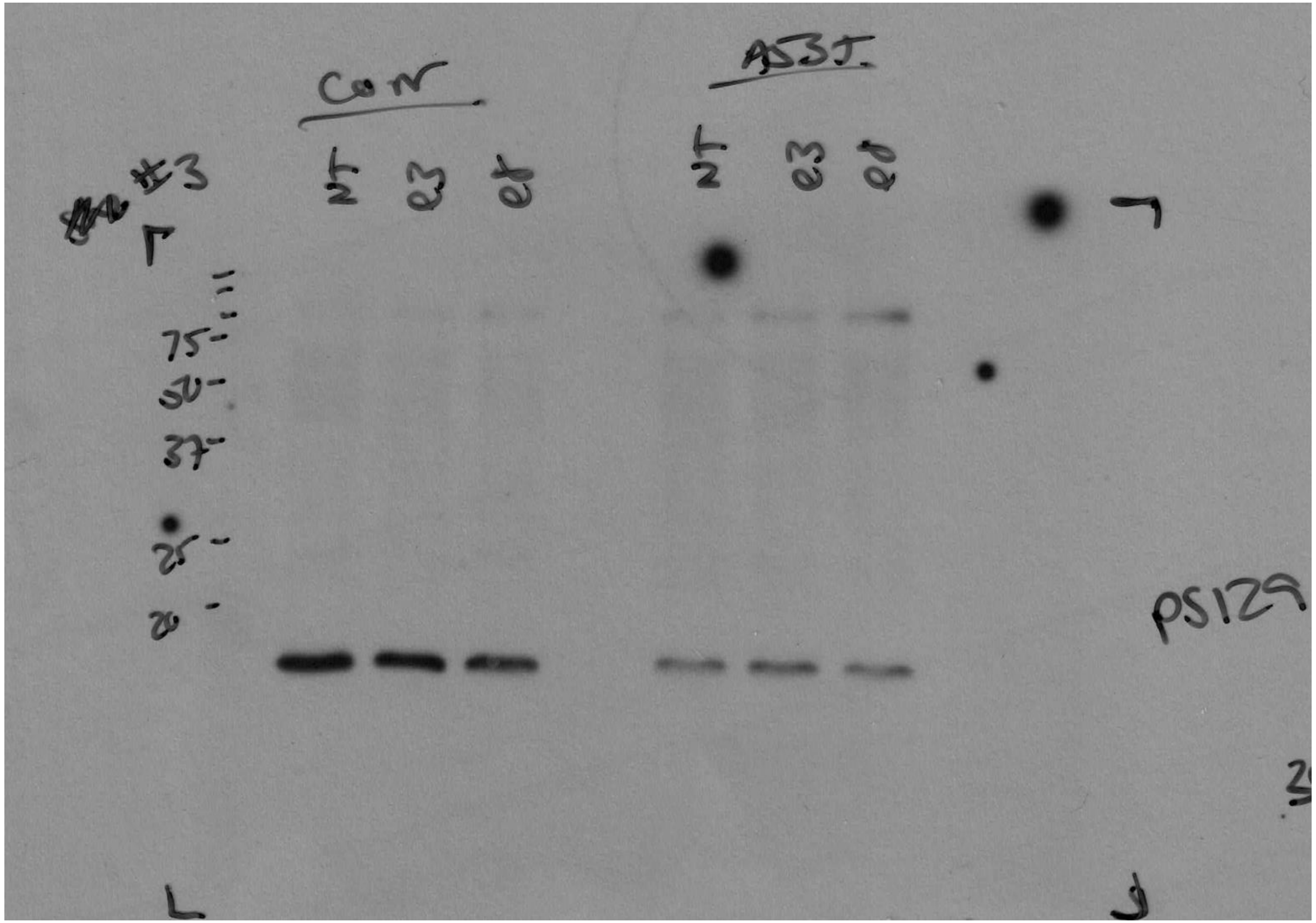

Fig3

a-syn n=3

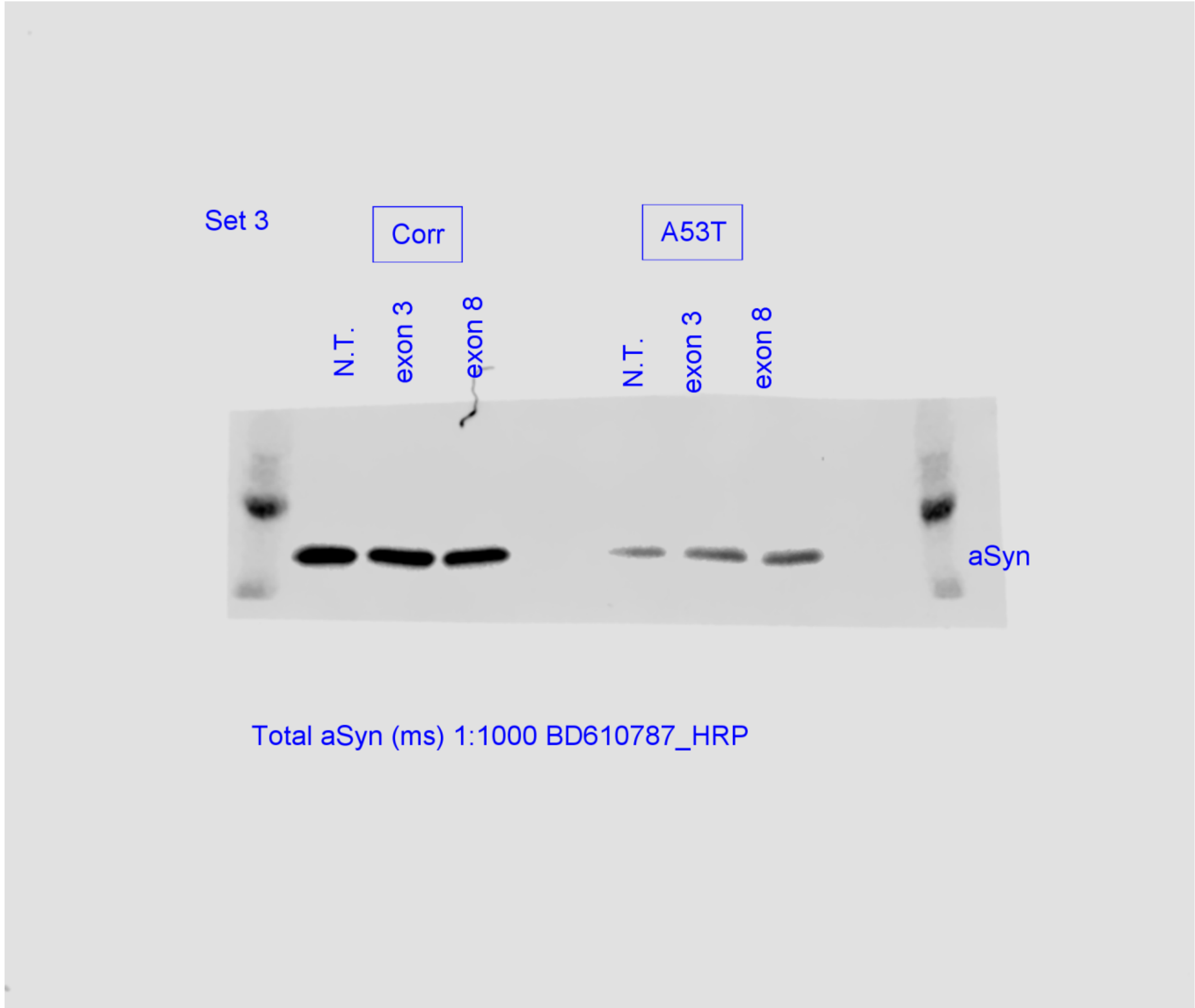

Set 3
Corr
A53T
N.T.
exon 3
exon 8
N.T.
exon 3
exon 8
aSyn
Total aSyn (ms) 1:1000 BD610787_HRP

Fig3

TH, beta-actin n=3

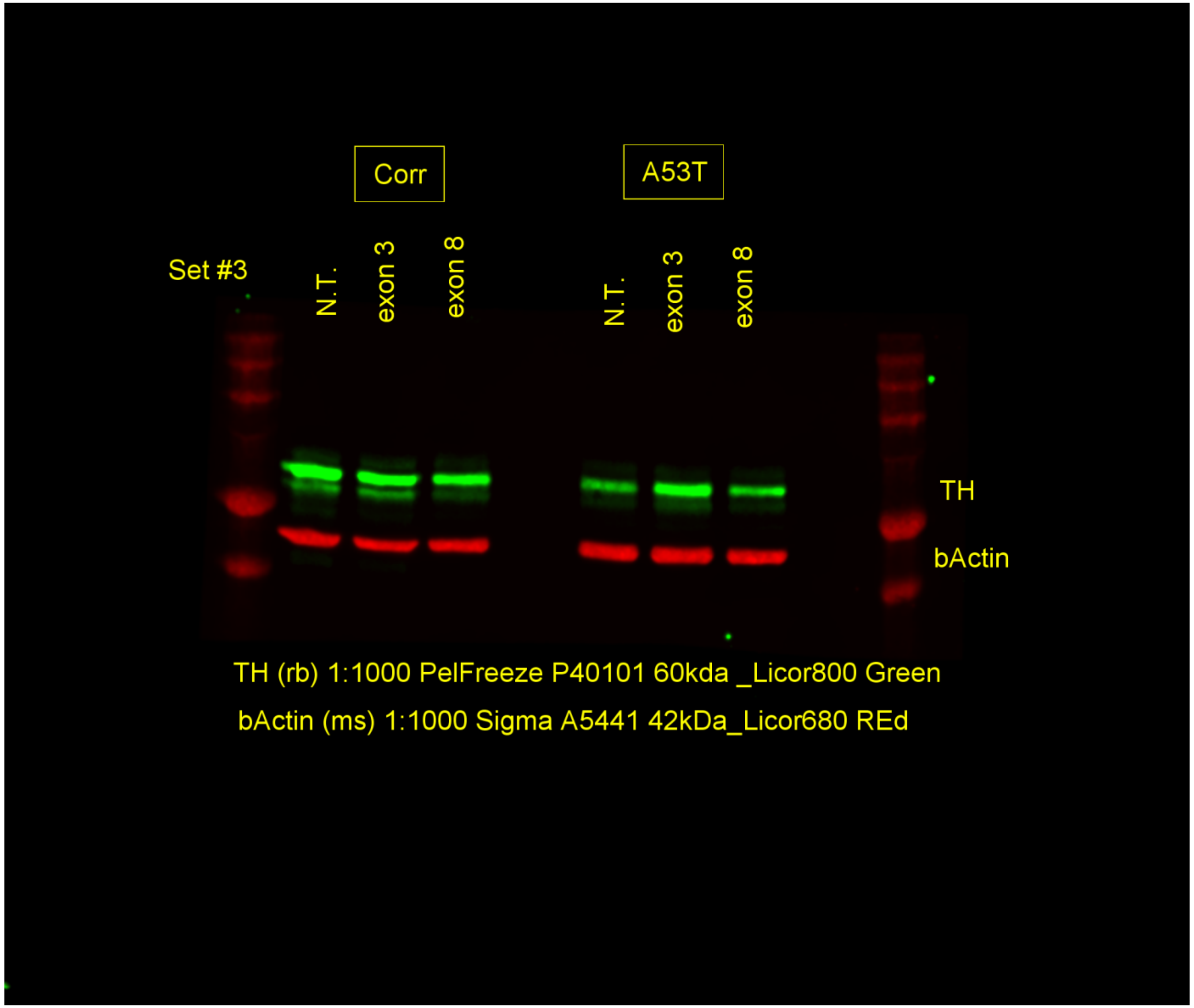

Fig4

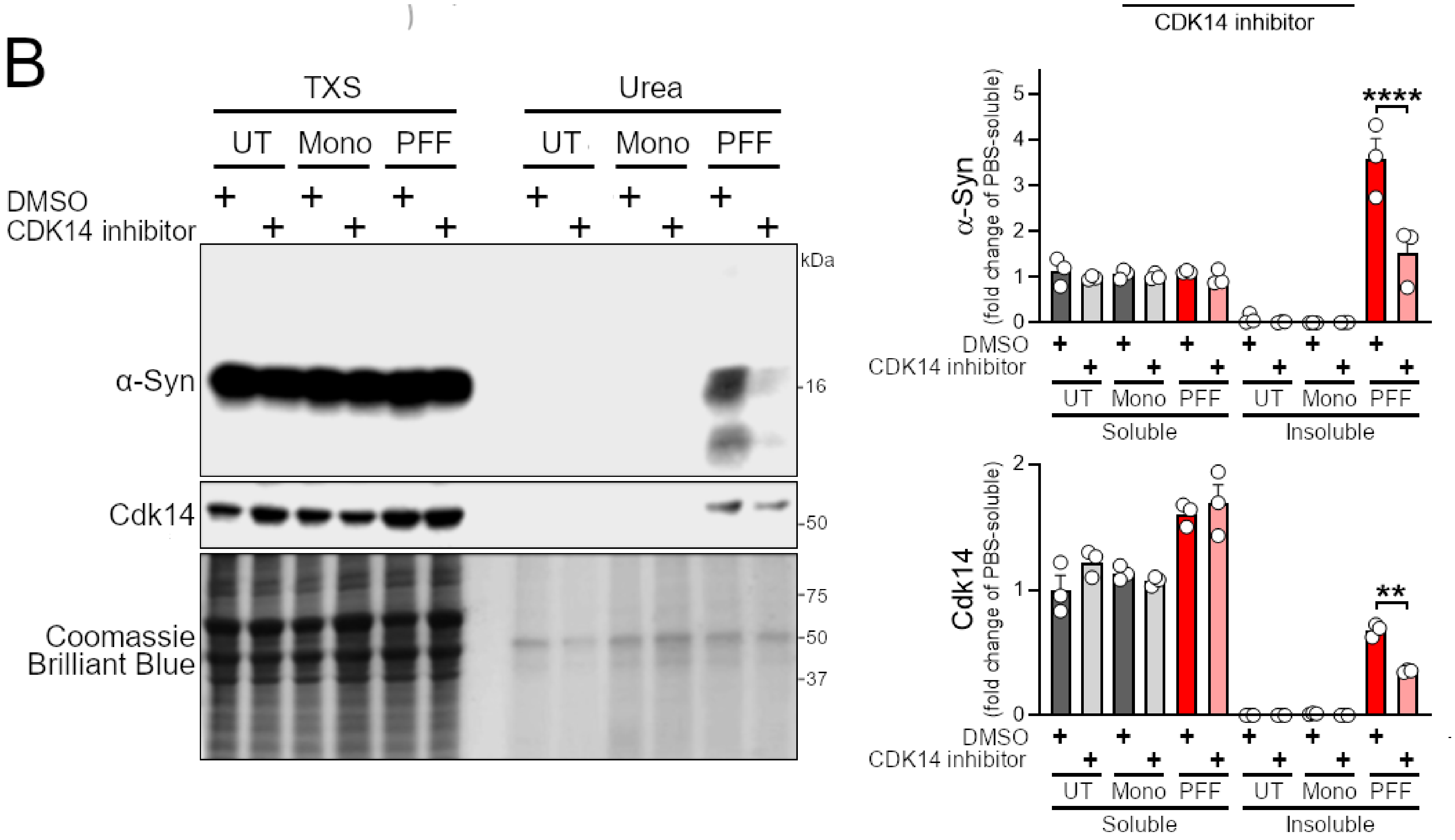

B
TXS
Urea
UT
Mono
PFF
DMSO
CDK14 inhibitor
kDa
α-Syn
Cdk14
Coomassie Brilliant Blue
16
50
75
37
CDK14 inhibitor
α-Syn (fold change of PBS-soluble)
Cdk14 (fold change of PBS-soluble)
****
**
Soluble
Insoluble

Fig4

a-syn n=1

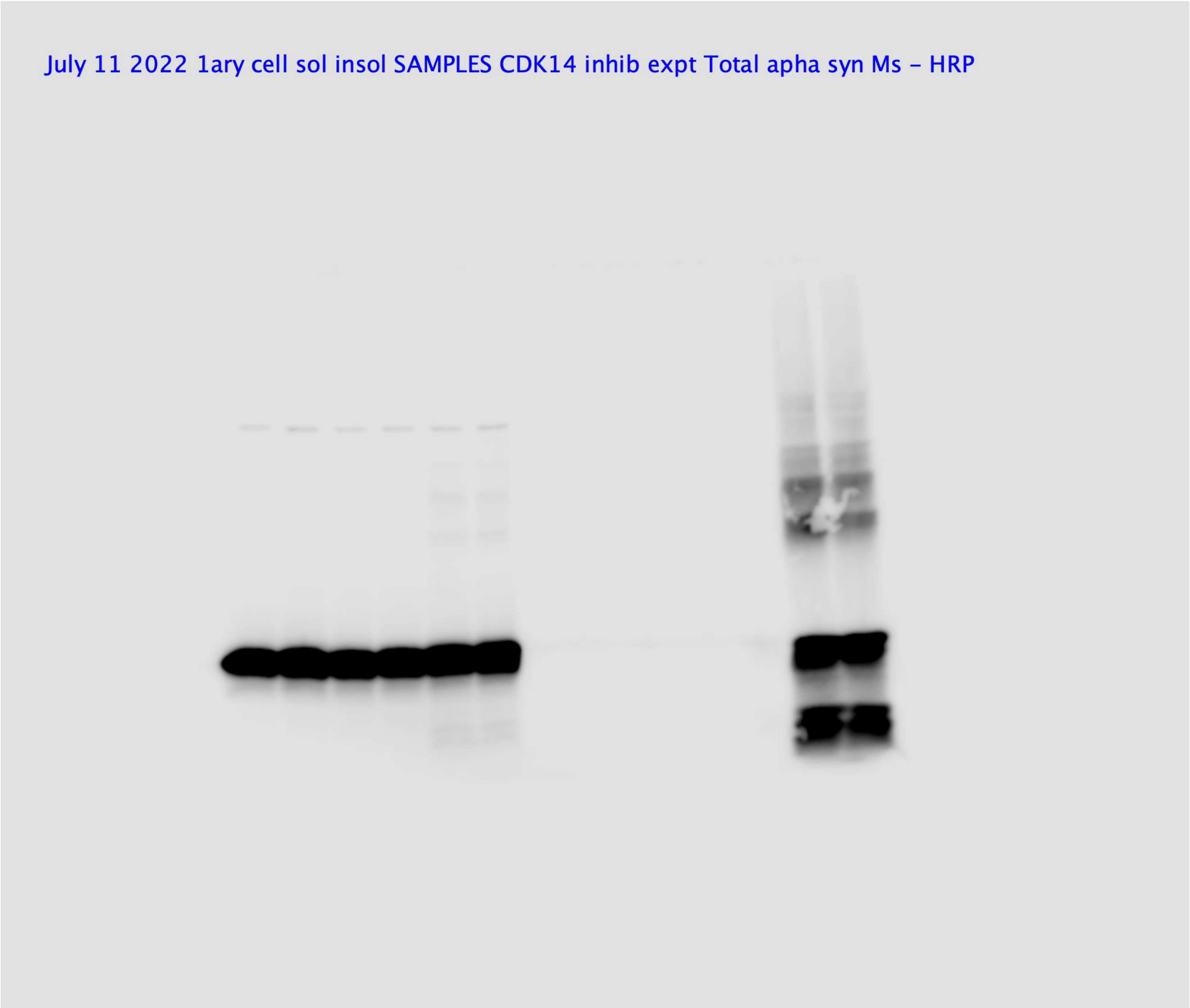
July 11 2022 1ary cell sol insol SAMPLES CDK14 inhib expt Total apha syn Ms - HRP

Fig4

CDK14 n=1

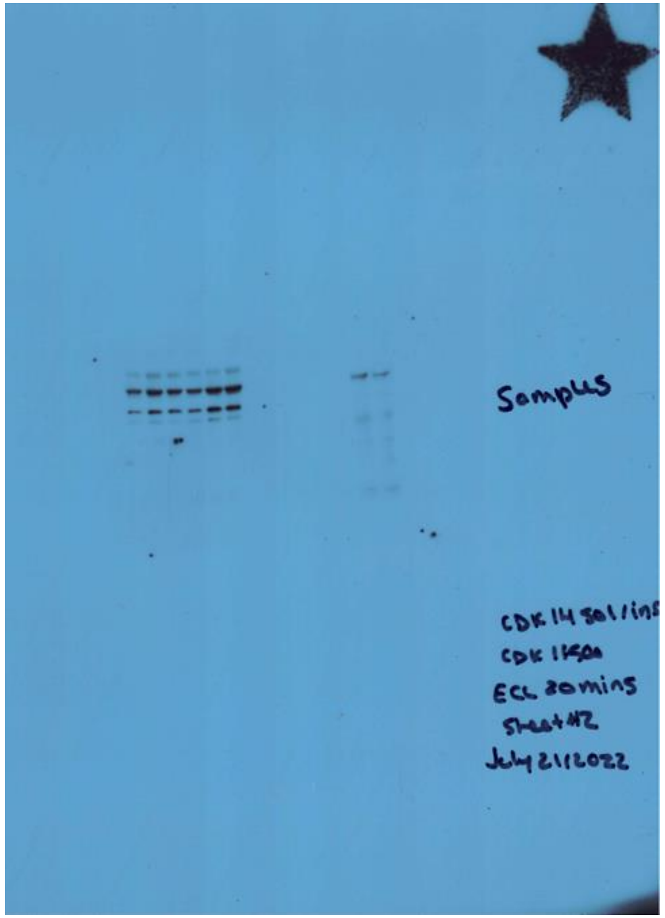

Fig4

Coomassie Brilliant B

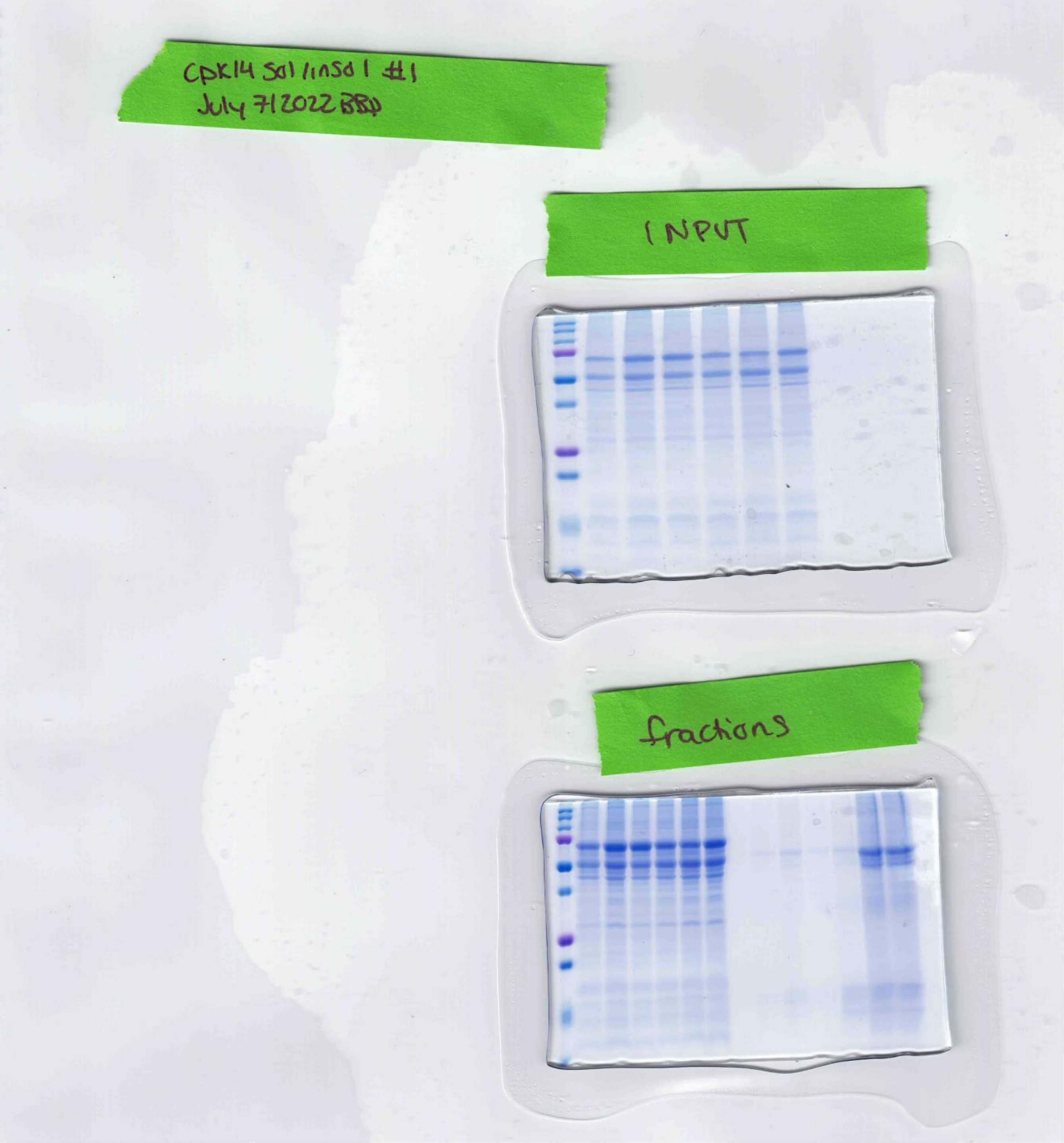

Fig4

a-syn n=2

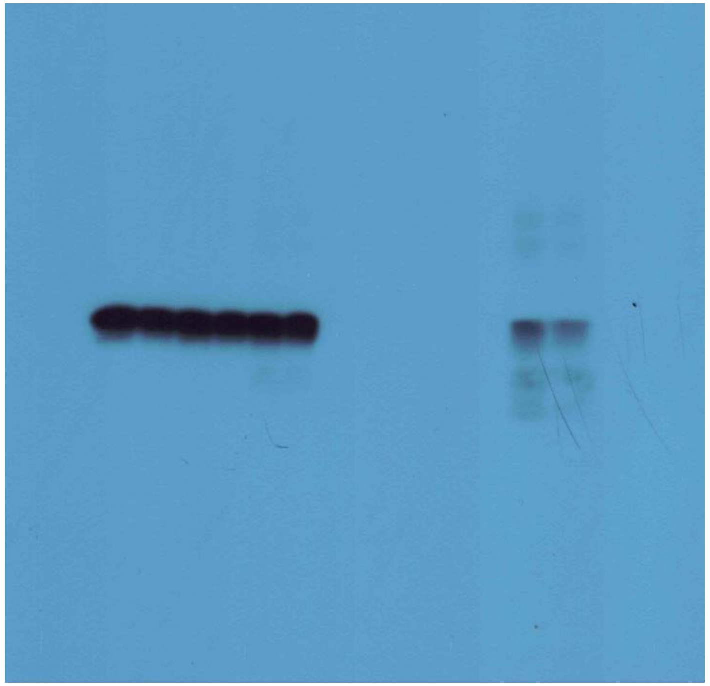

Fig4

CDK14 n=2

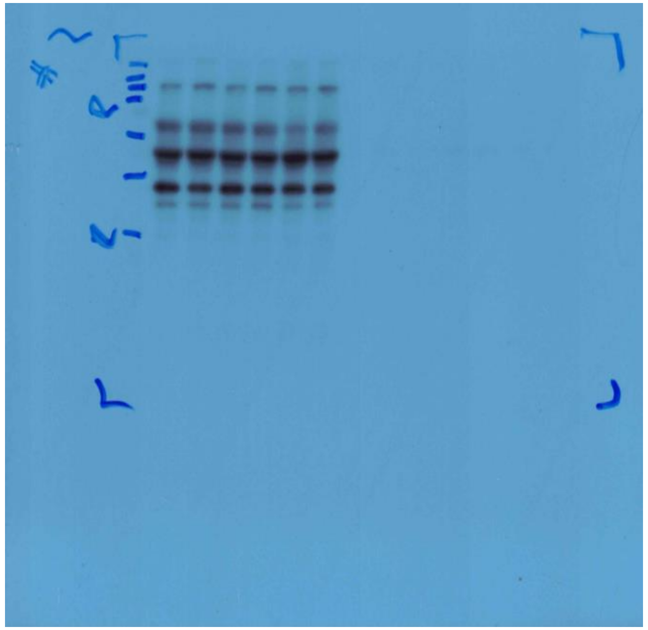

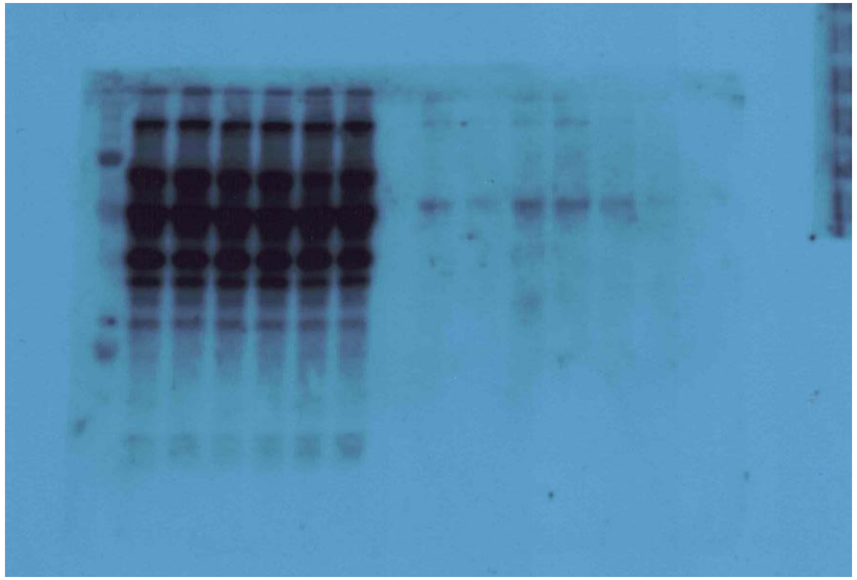

Fig4

Coomassie Brilliant Blue n=2

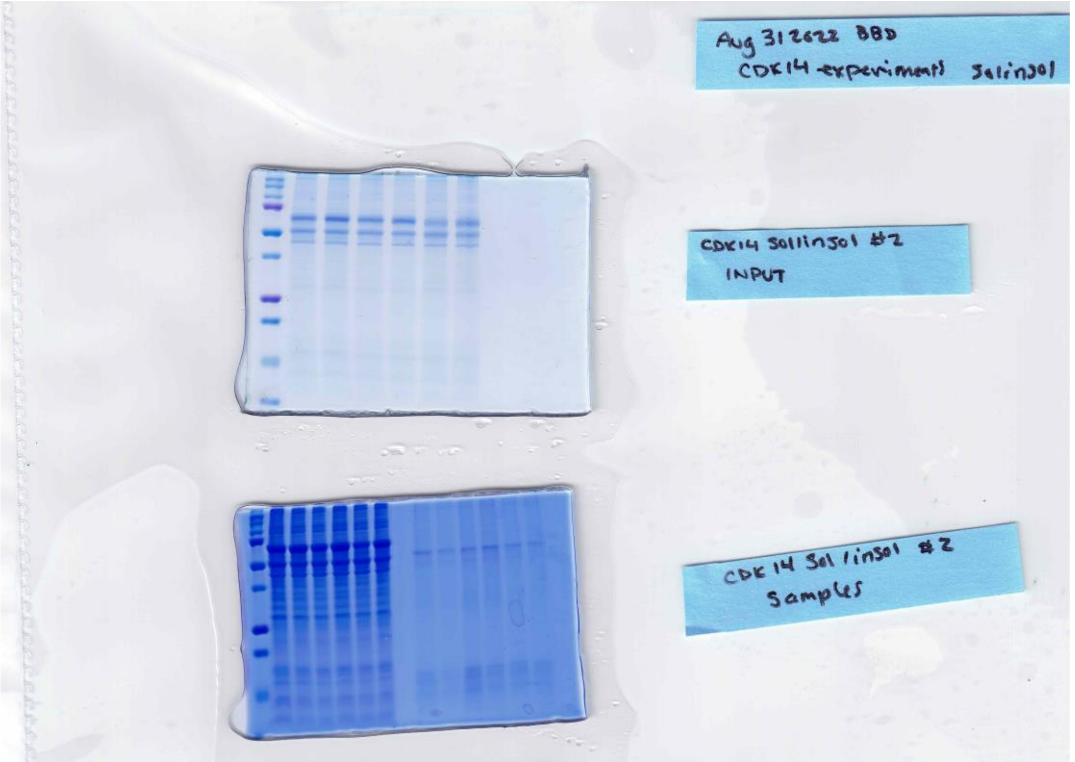

Fig4

a-syn n=3

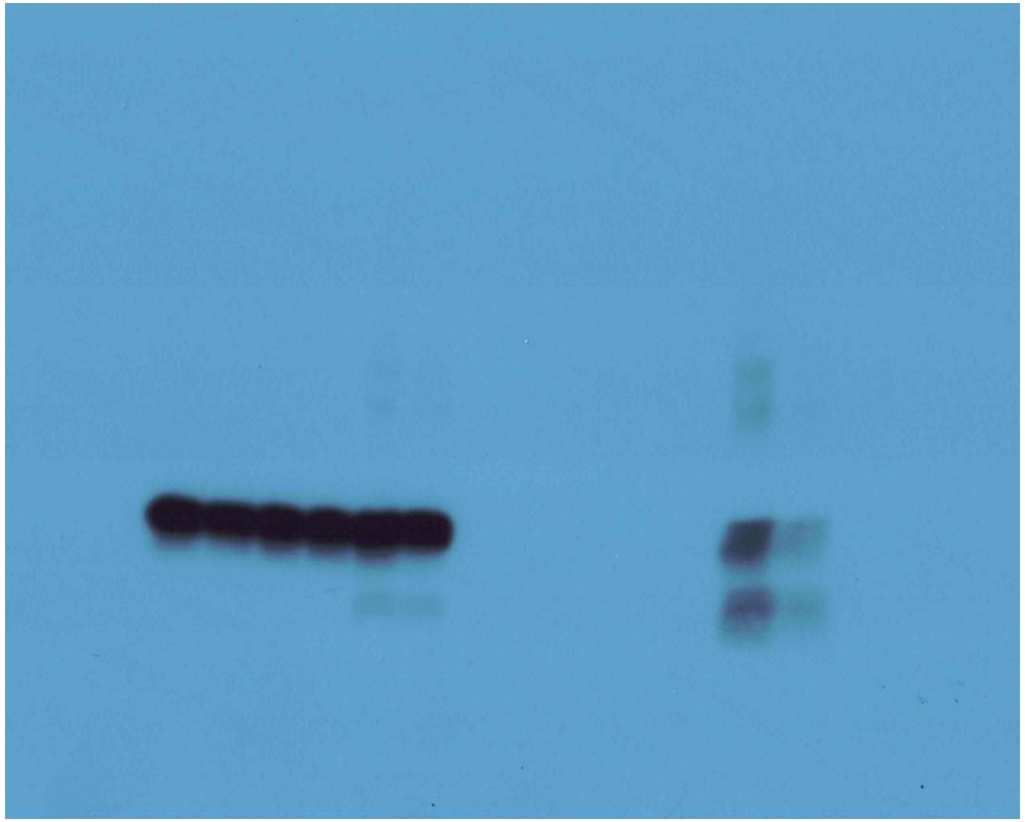

Fig4

CDK14 n=3

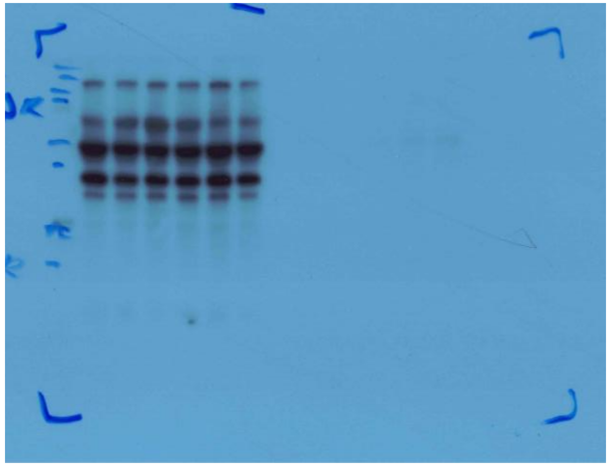

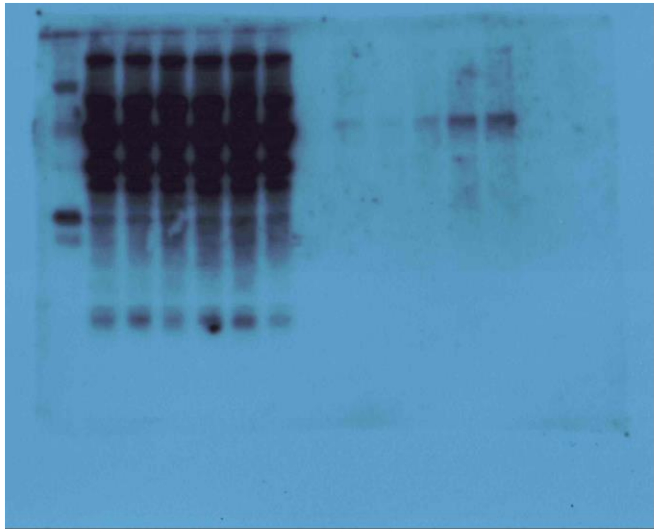

Fig4

Coomassie Brilliant Blue n=3

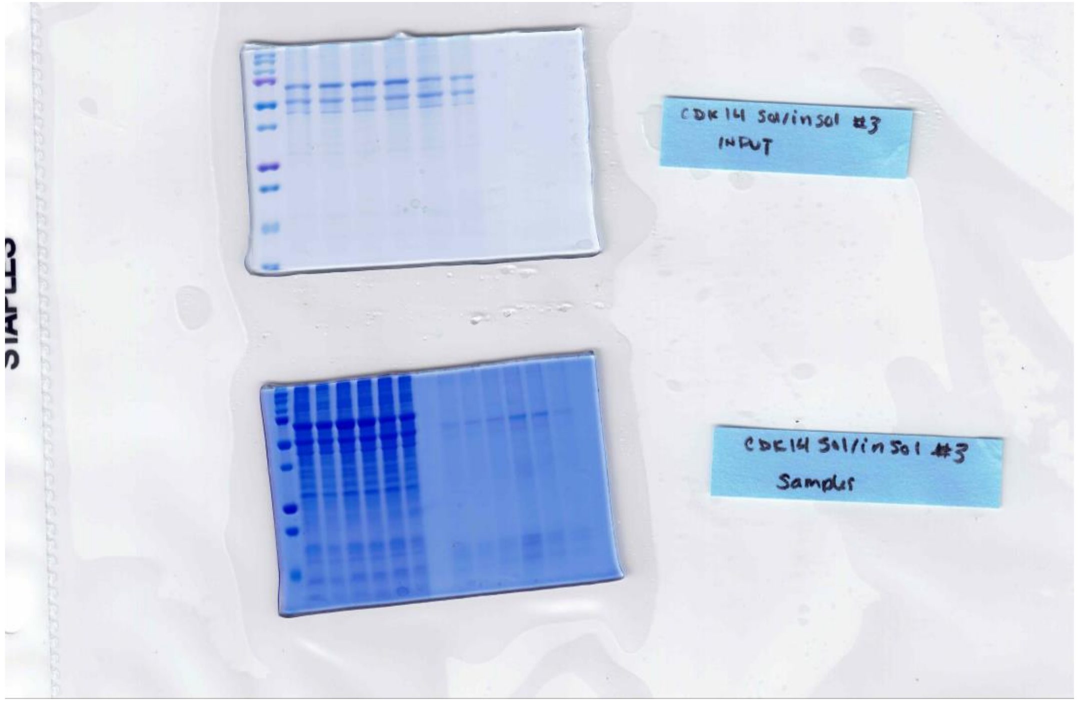

Fig5

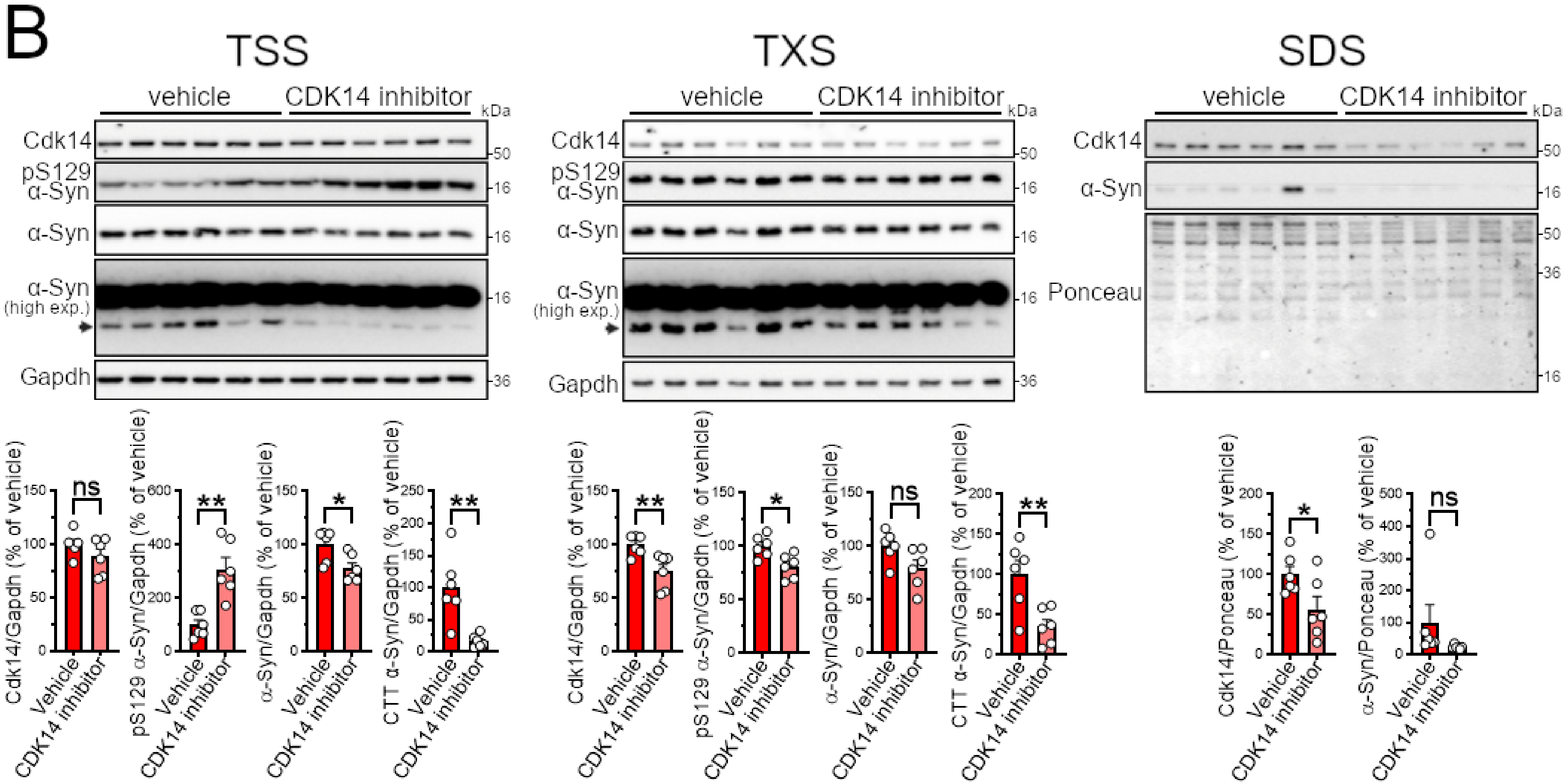
B
TSS
TXS
SDS
vehicle
CDK14 inhibitor
kDa
Cdk14
pS129 α-Syn
α-Syn
α-Syn (high exp.)
Gapdh
Ponceau
50
16
36
Cdk14/Gapdh (% of vehicle)
pS129 α-Syn/Gapdh (% of vehicle)
α-Syn/Gapdh (% of vehicle)
CTT α-Syn/Gapdh (% of vehicle)
Cdk14/Ponceau (% of vehicle)
α-Syn/Ponceau (% of vehicle)
Vehicle
CDK14 inhibitor
ns
*
**

Fig5

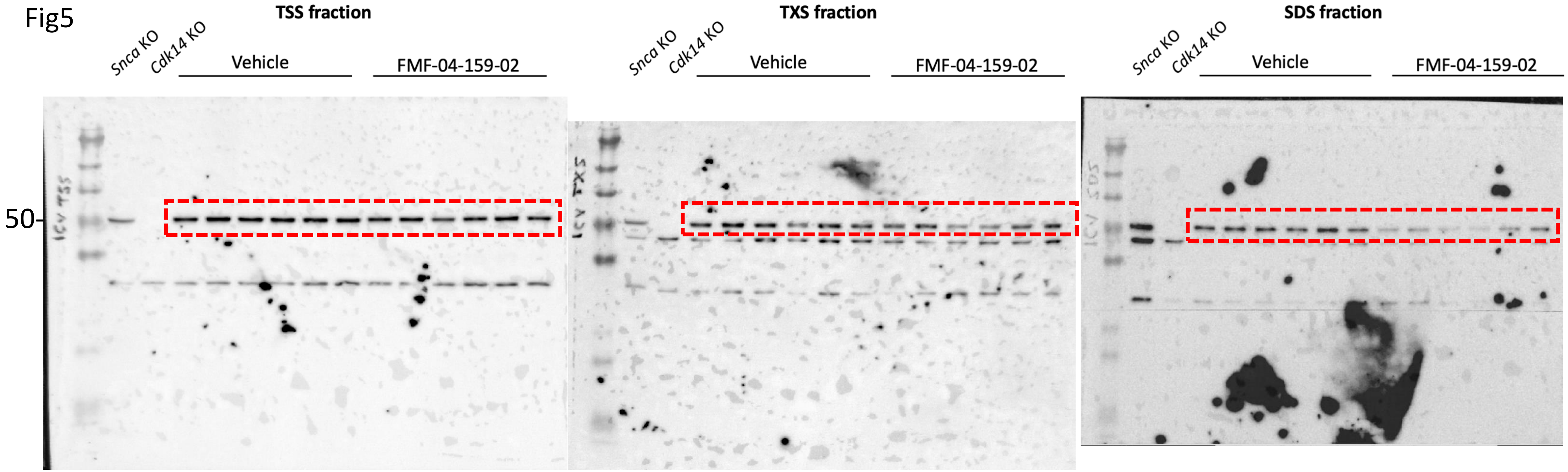
TSS fraction
TXS fraction
SDS fraction
Snca KO
Cdk14 KO
Vehicle
FMF-04-159-02
50-

CDK14

Fig5

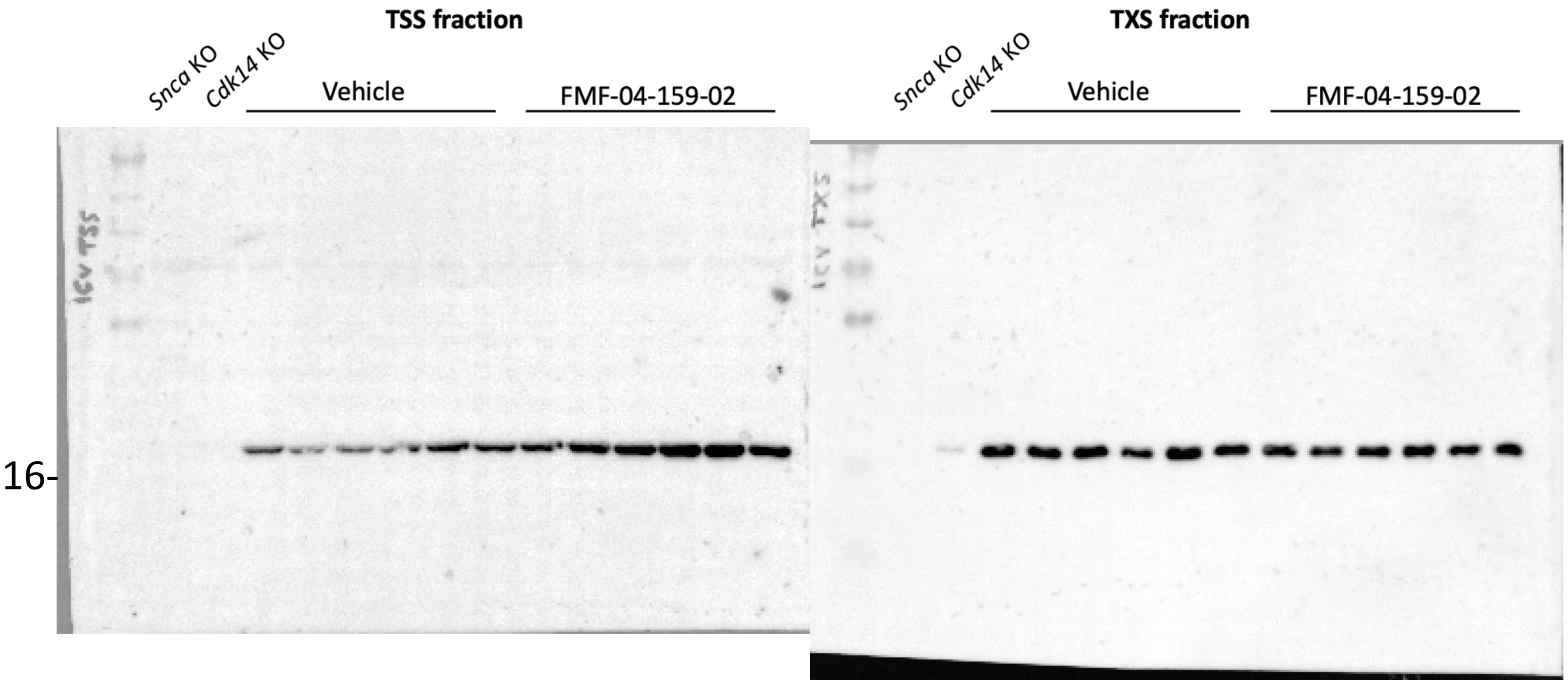

pS129 α-Syn

Fig5

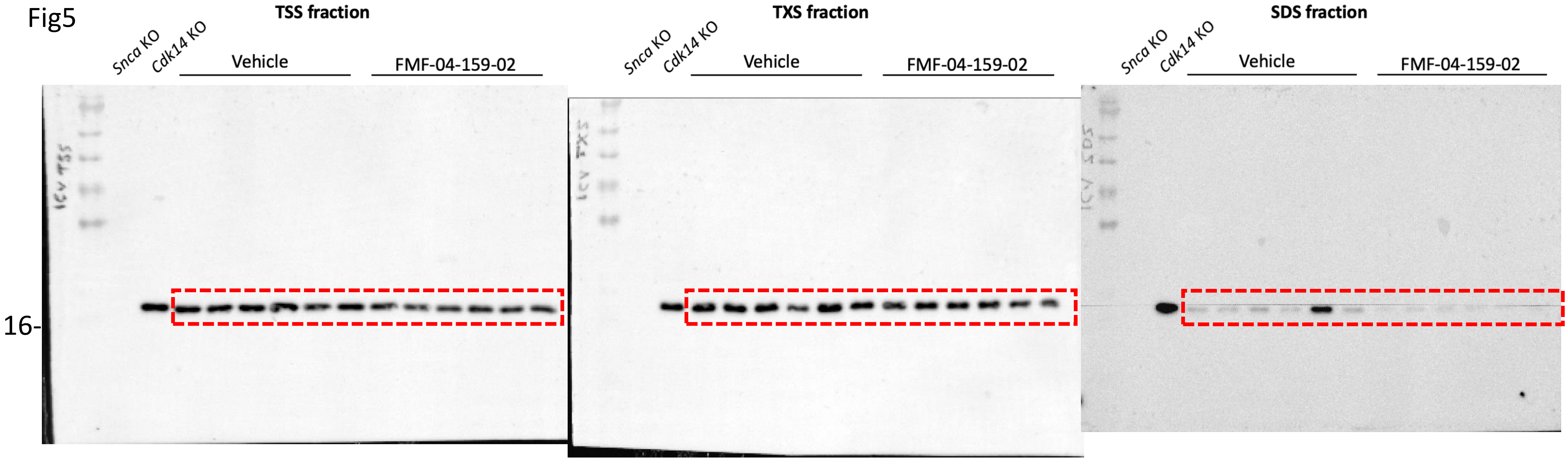
TSS fraction
Snca KO
Cdk14 KO
Vehicle
FMF-04-159-02
16-
TXS fraction
Snca KO
Cdk14 KO
Vehicle
FMF-04-159-02
SDS fraction
Snca KO
Cdk14 KO
Vehicle
FMF-04-159-02

α-Syn

Fig5

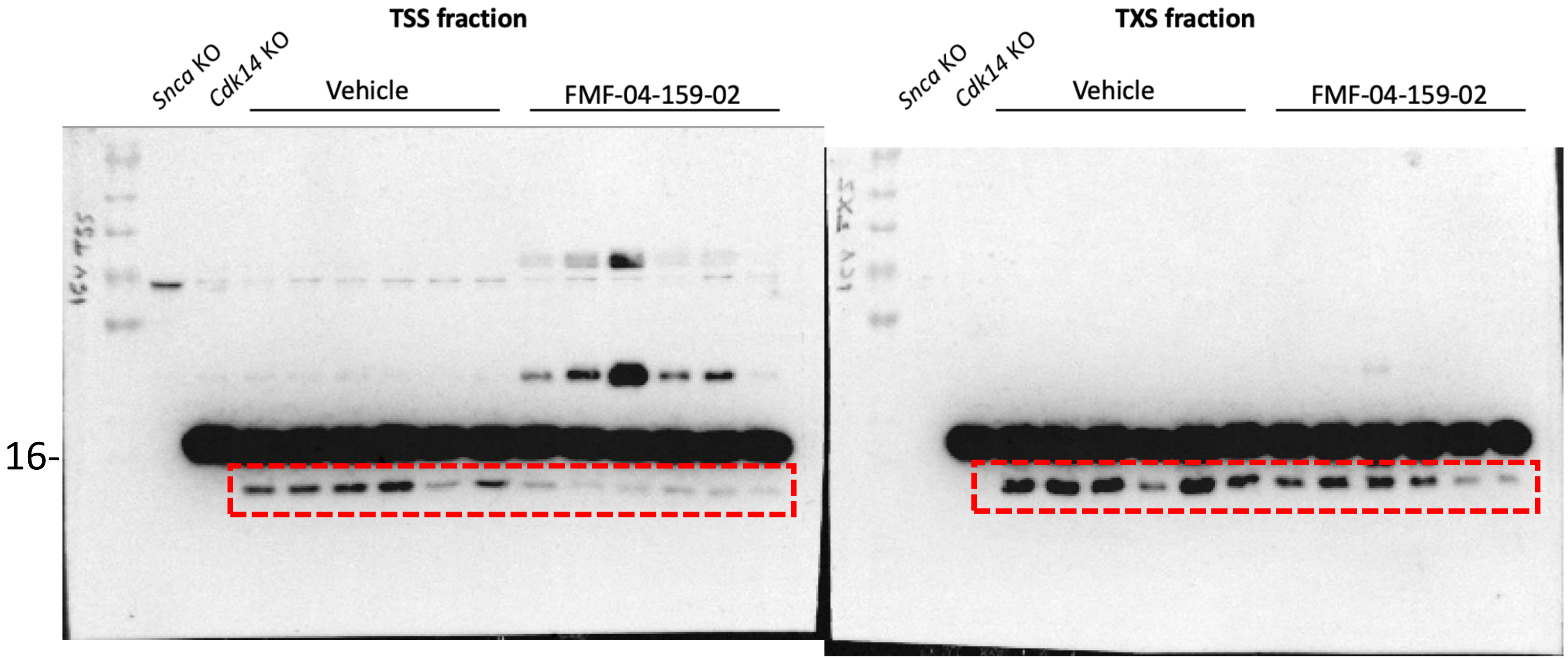

α-Syn high exposure

Fig5

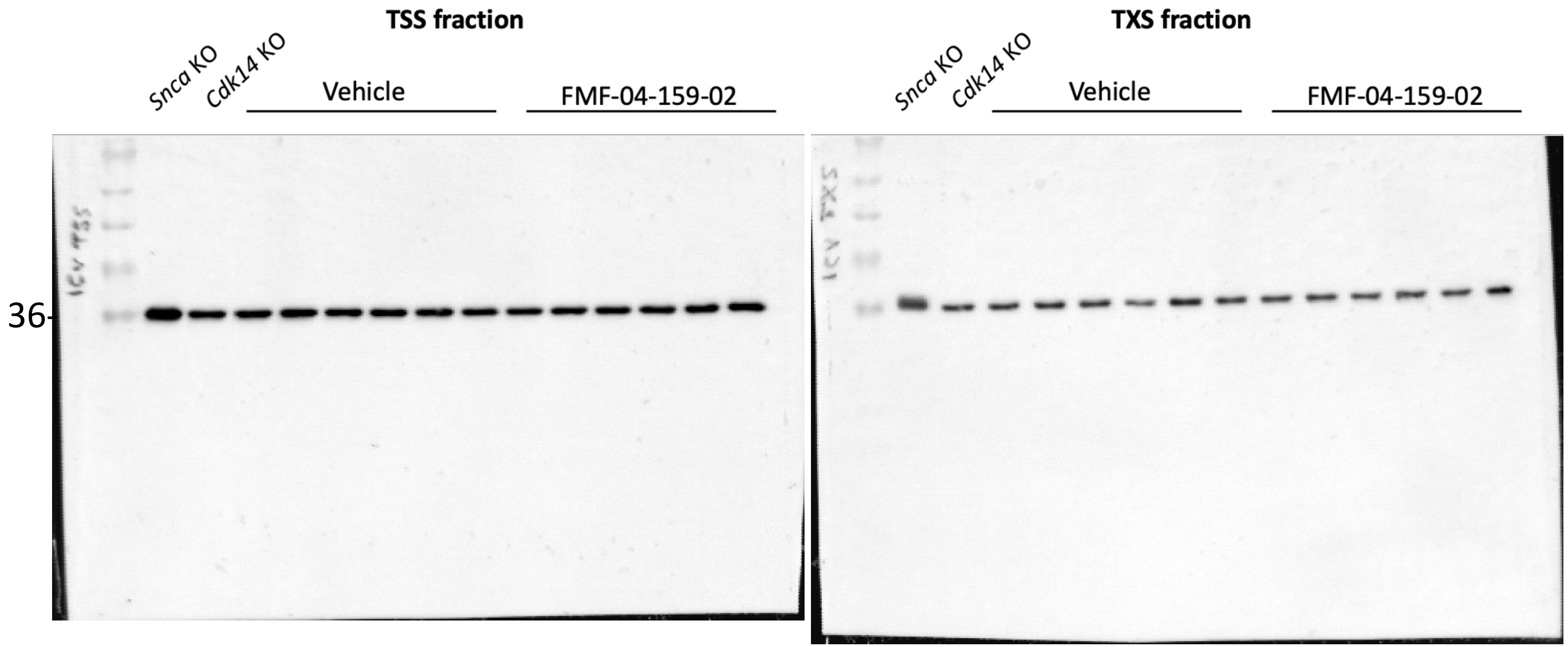
TSS fraction
Snca KO
Cdk14 KO
Vehicle
FMF-04-159-02
36
TXS fraction
Snca KO
Cdk14 KO
Vehicle
FMF-04-159-02

GAPDH

Fig5

SDS Ponceau S

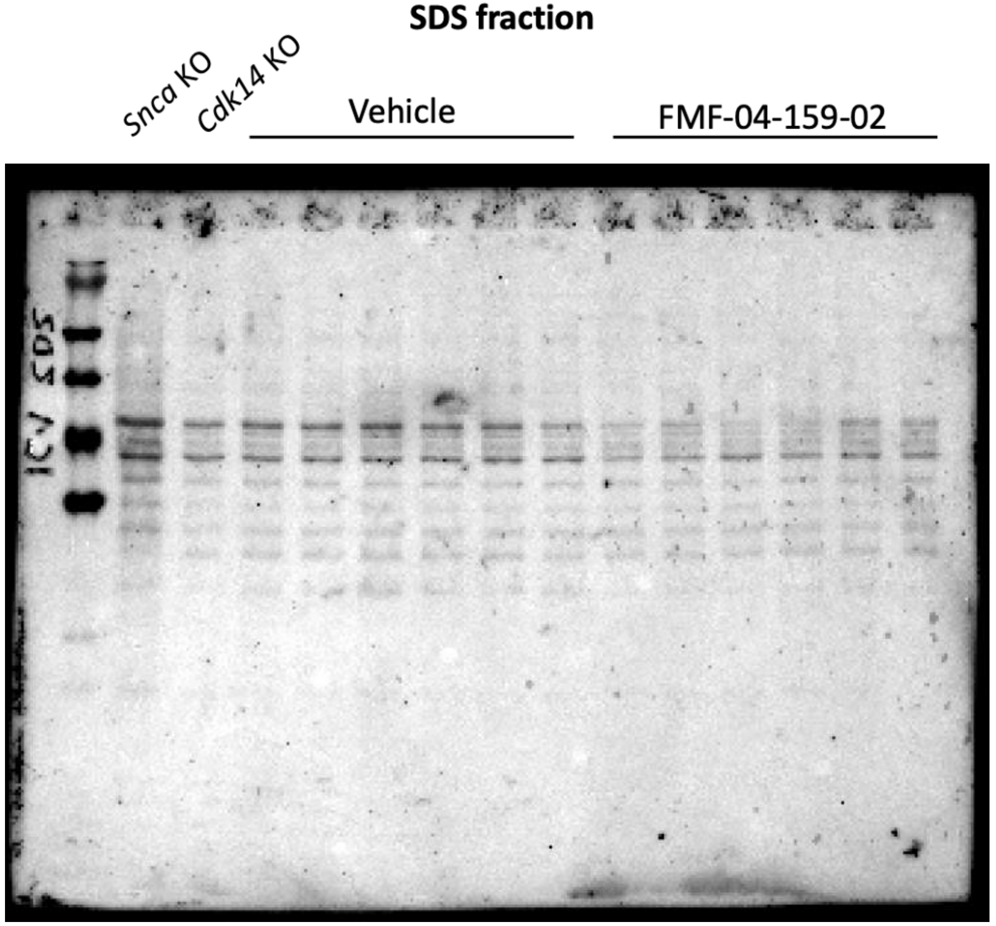

Fig S1B

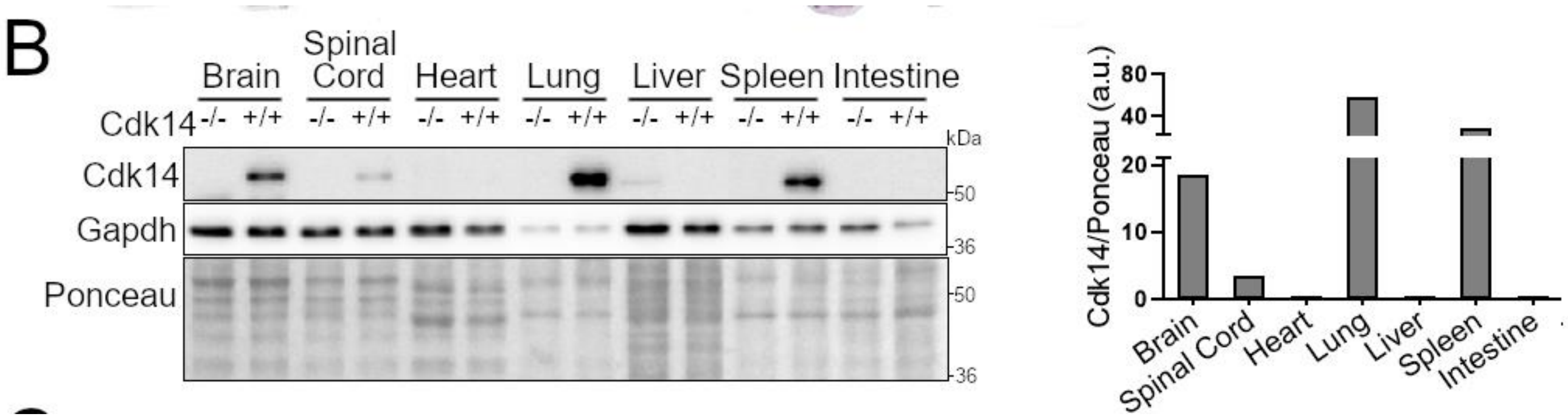
B
Brain
Spinal Cord
Heart
Lung
Liver
Spleen
Intestine
Cdk14
-/- +/+ -/- +/+ -/- +/+ -/- +/+ -/- +/+ -/- +/+ -/- +/+
kDa
Cdk14
Gapdh
Ponceau
50
36
50
36
Cdk14/Ponceau (a.u.)
80
40
20
10
0
Brain
Spinal Cord
Heart
Lung
Liver
Spleen
Intestine

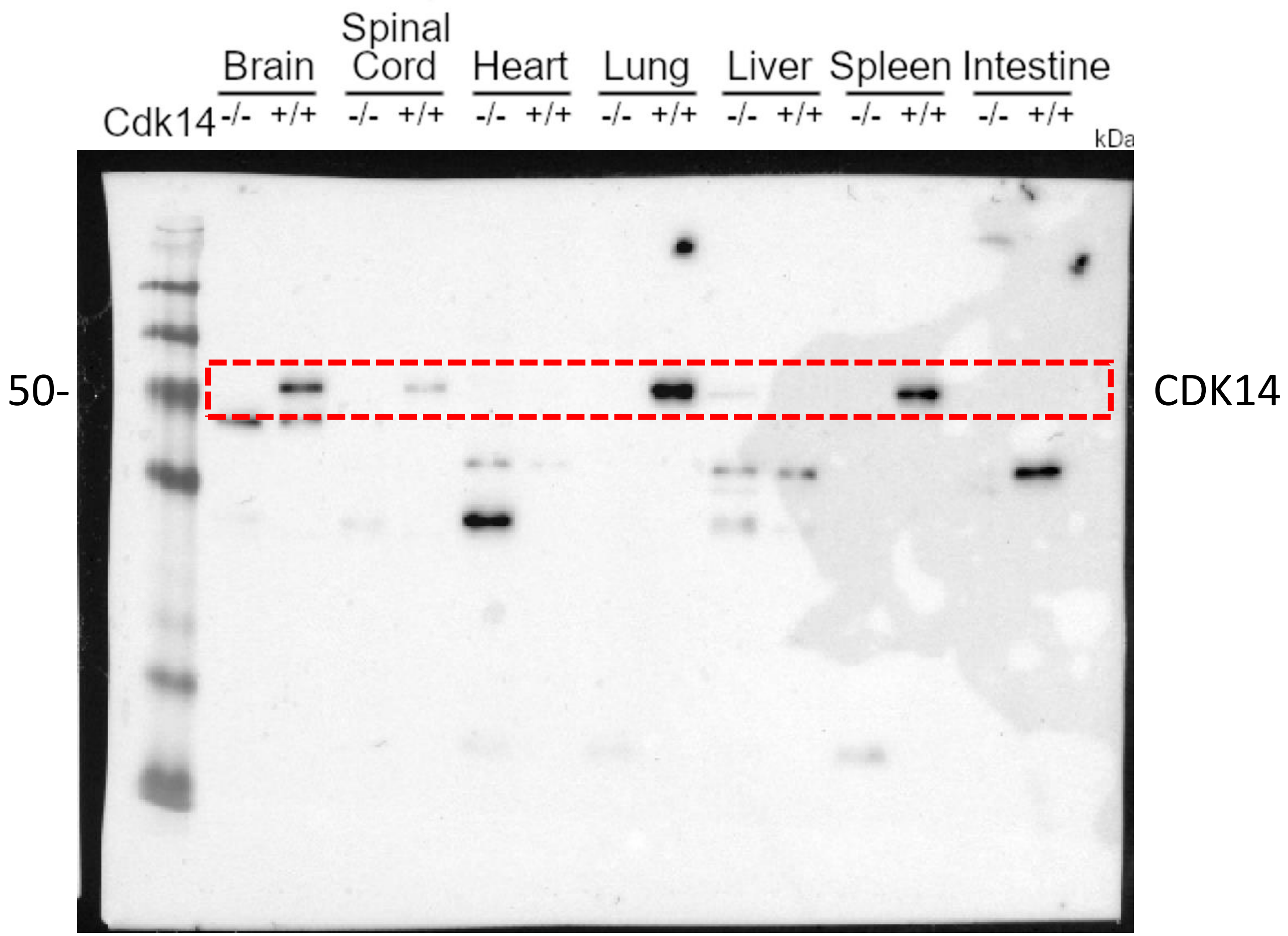
Brain
Spinal Cord
Heart
Lung
Liver
Spleen
Intestine
Cdk14
-/- +/+ -/- +/+ -/- +/+ -/- +/+ -/- +/+ -/- +/+ -/- +/+
kDa
50-
CDK14

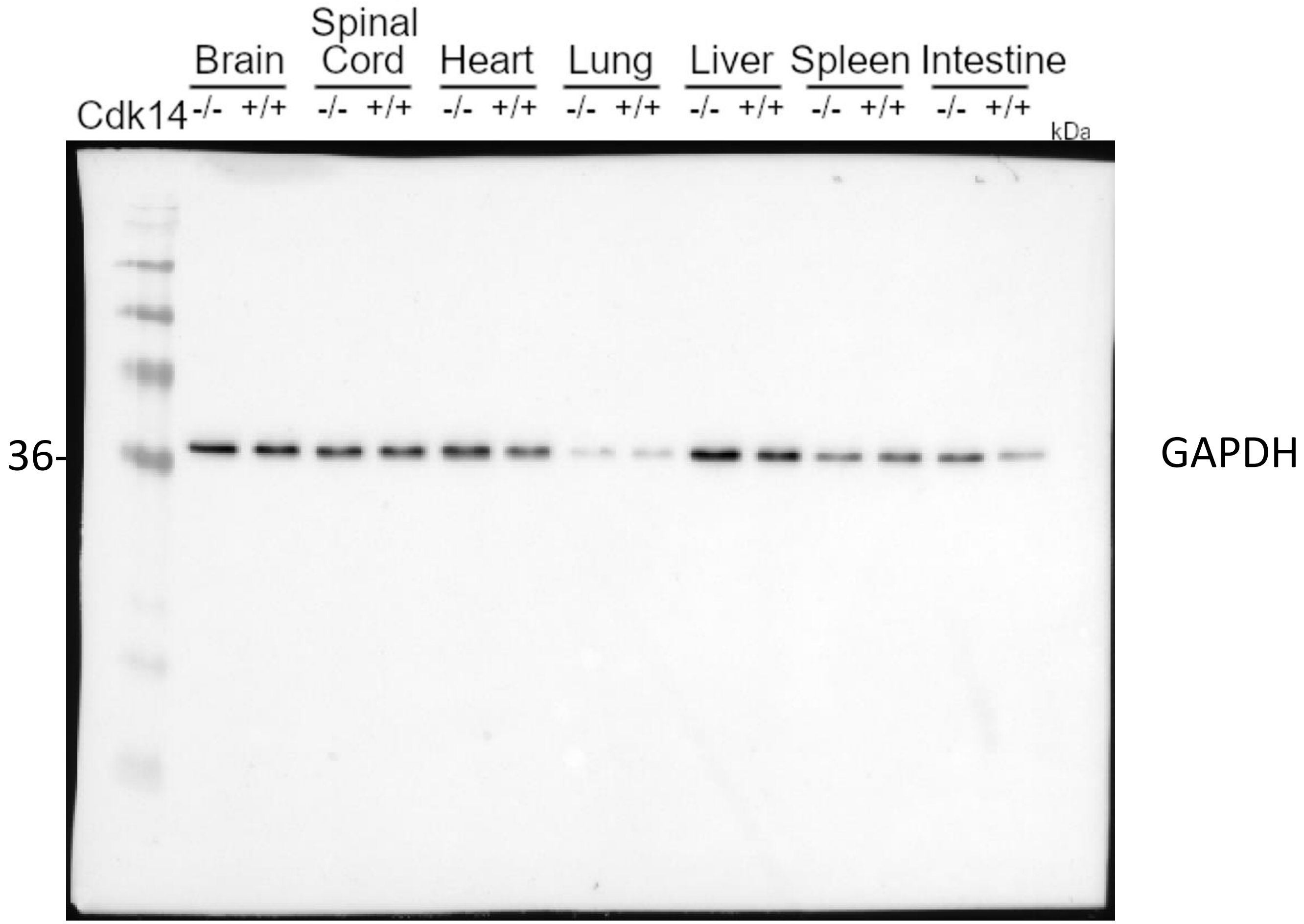
Spinal
Brain
Cord
Heart
Lung
Liver
Spleen
Intestine
Cdk14
-/-
+/+
kDa
36-
GAPDH

Ponceau

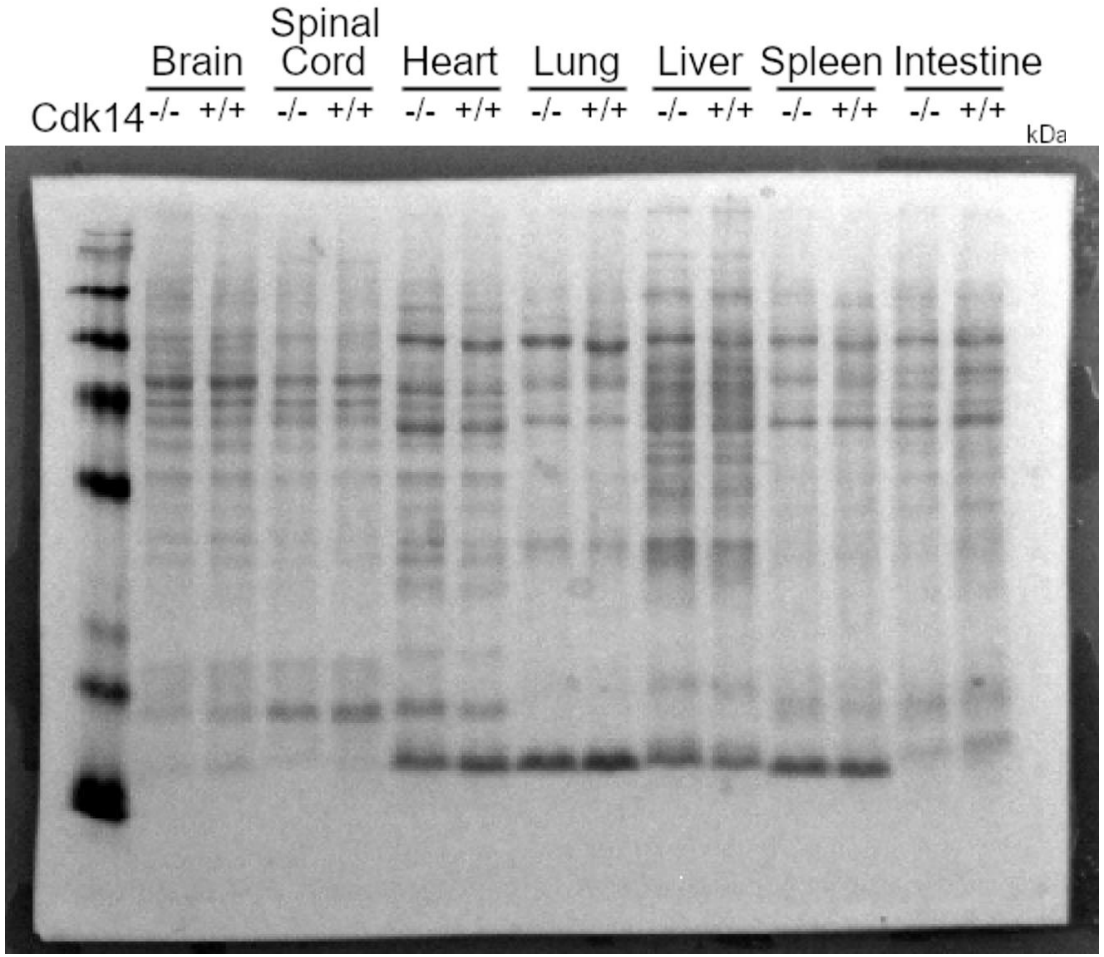
Brain
Spinal Cord
Heart
Lung
Liver
Spleen
Intestine
Cdk14
-/- +/+ -/- +/+ -/- +/+ -/- +/+ -/- +/+ -/- +/+ -/- +/+
kDa

Fig S2E

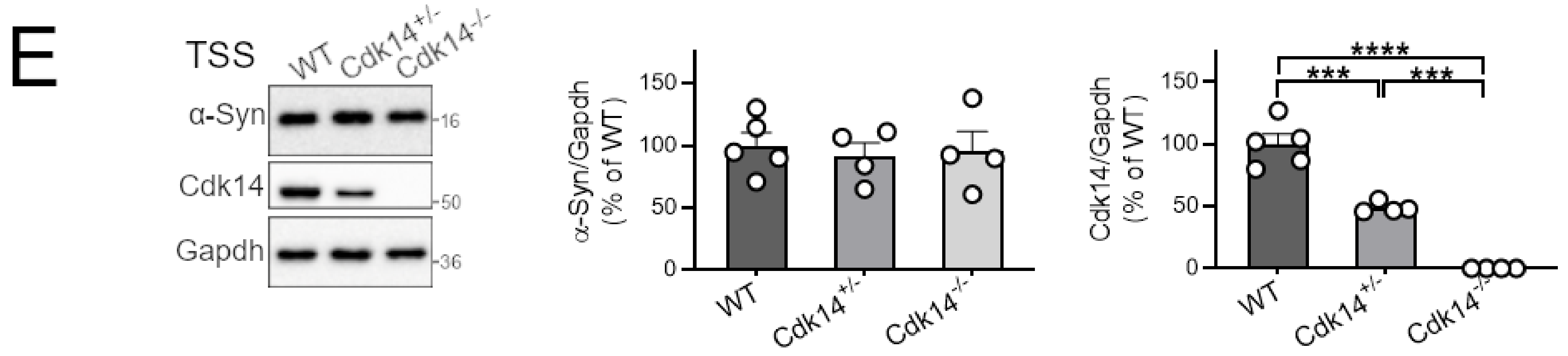
E
TSS
WT
Cdk14+/-
Cdk14-/-
α-Syn
Cdk14
Gapdh
16
50
36
α-Syn/Gapdh (% of WT)
150
100
50
0
WT
Cdk14+/-
Cdk14-/-
Cdk14/Gapdh (% of WT)
****
***
***

Fig S2E

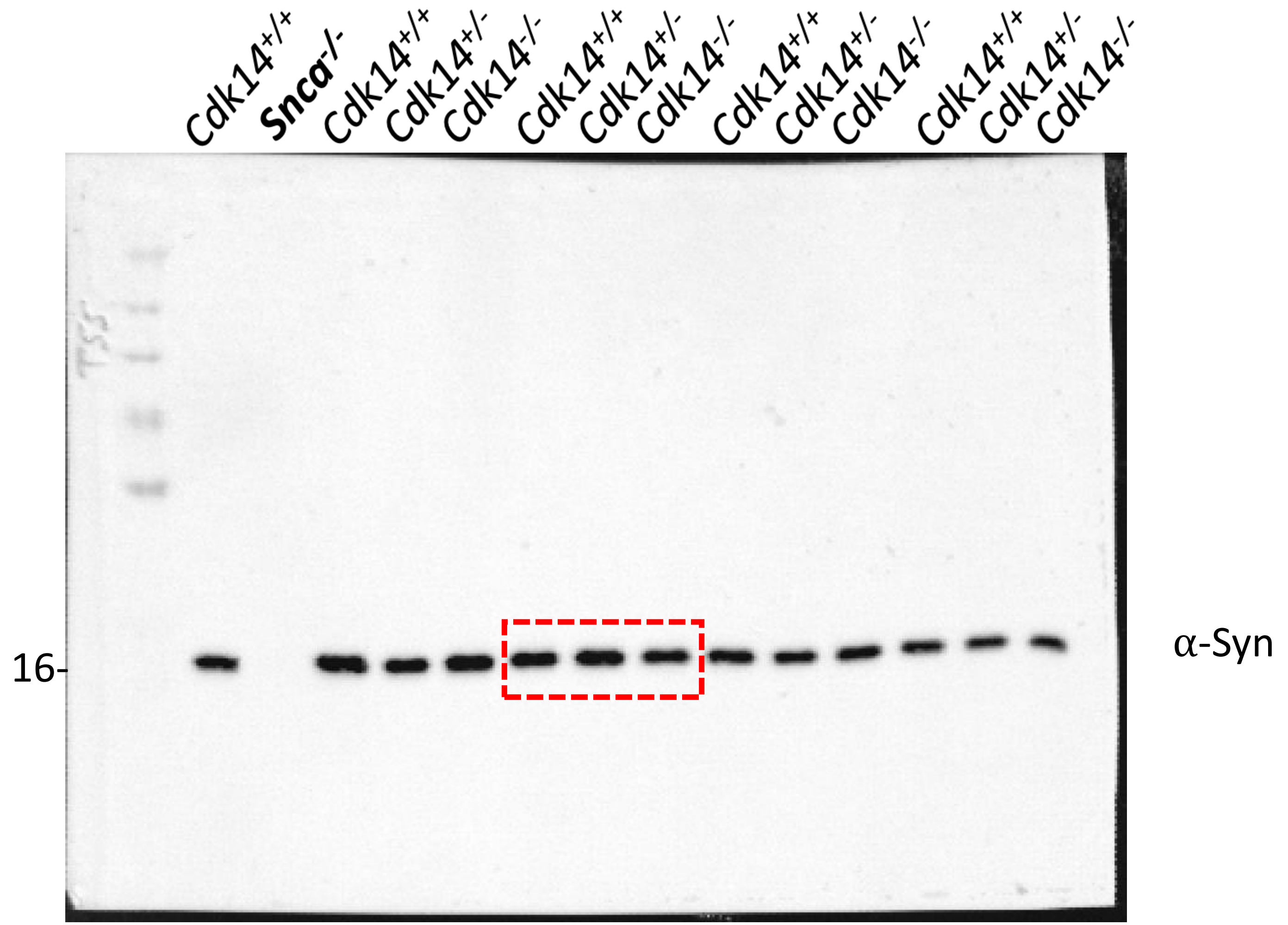

Cdk14+/+
Snca-/-
Cdk14+/+
Cdk14+/-
Cdk14-/-
Cdk14+/+
Cdk14+/-
Cdk14-/-
Cdk14+/+
Cdk14+/-
Cdk14-/-
Cdk14+/+
Cdk14+/-
Cdk14-/-
16-
α-Syn

Fig S2E

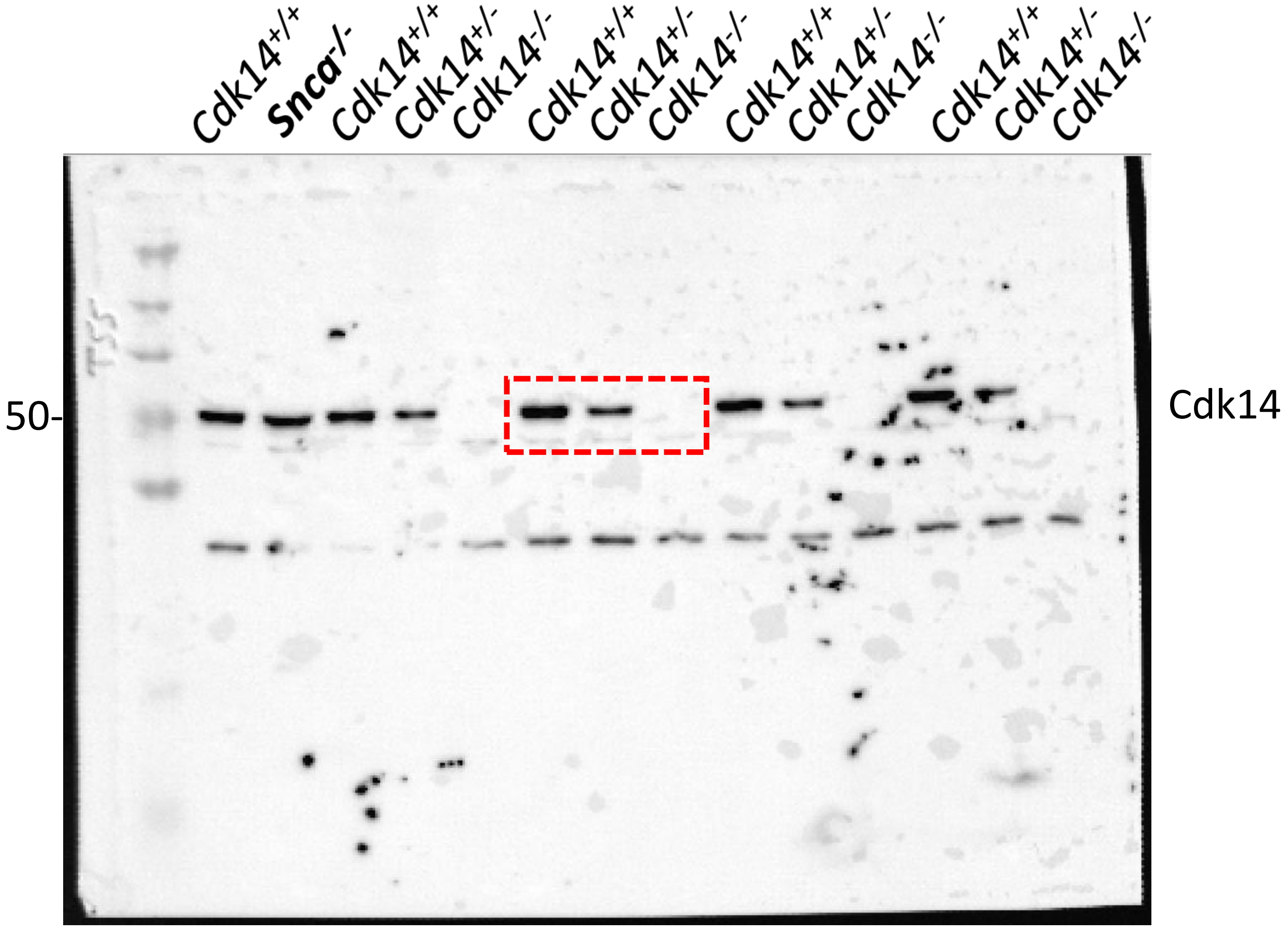

Cdk14+/+
Snca-/-
Cdk14+/+
Cdk14+/-
Cdk14-/-
Cdk14+/+
Cdk14+/-
Cdk14-/-
Cdk14+/+
Cdk14+/-
Cdk14-/-
Cdk14+/+
Cdk14+/-
Cdk14-/-
50-
Cdk14

Fig S2E

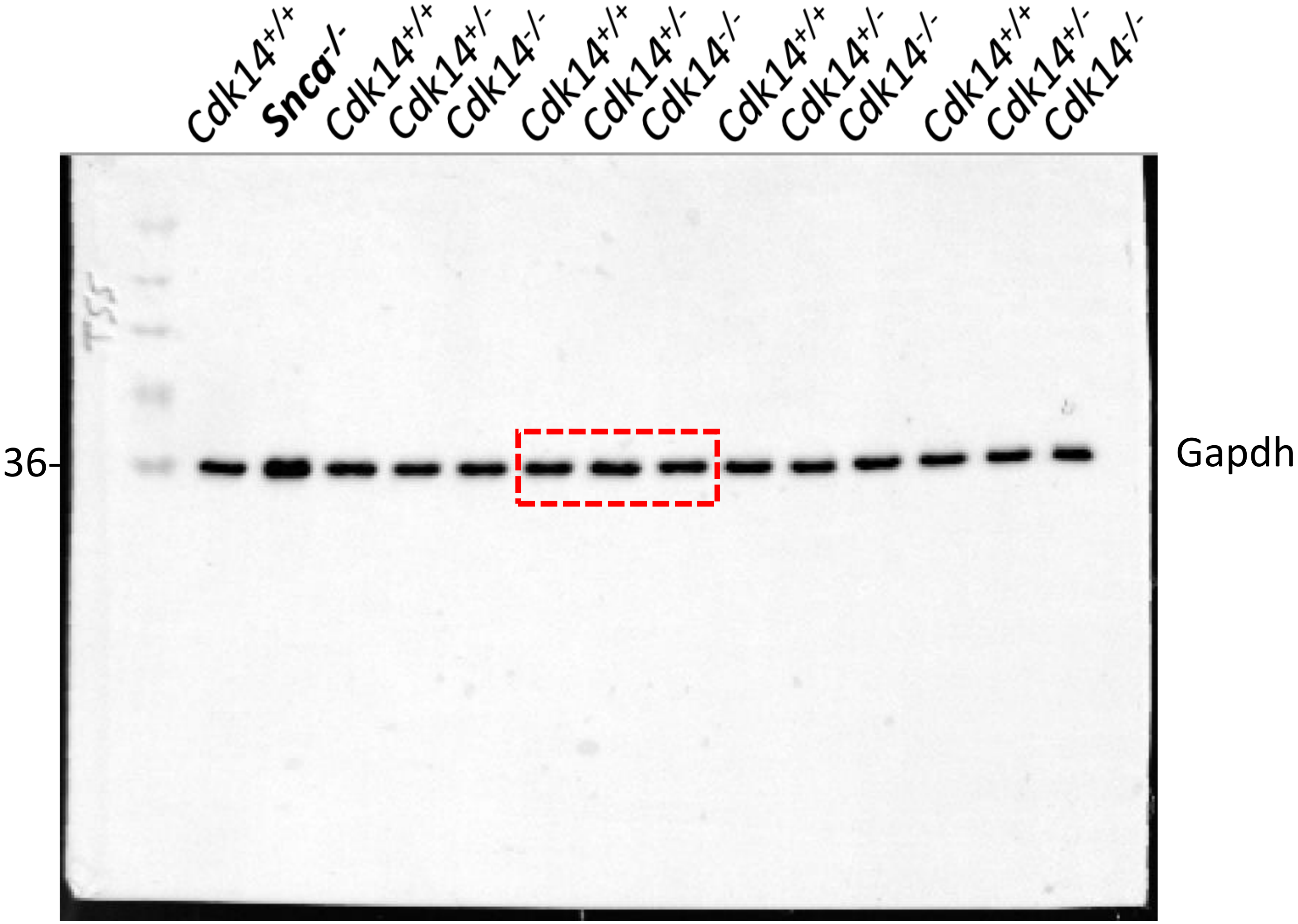
Cdk14+/+
Snca-/-
Cdk14+/+
Cdk14+/-
Cdk14-/-
Cdk14+/+
Cdk14+/-
Cdk14-/-
Cdk14+/+
Cdk14+/-
Cdk14-/-
Cdk14+/+
Cdk14+/-
Cdk14-/-
36-
Gapdh

Fig S3A

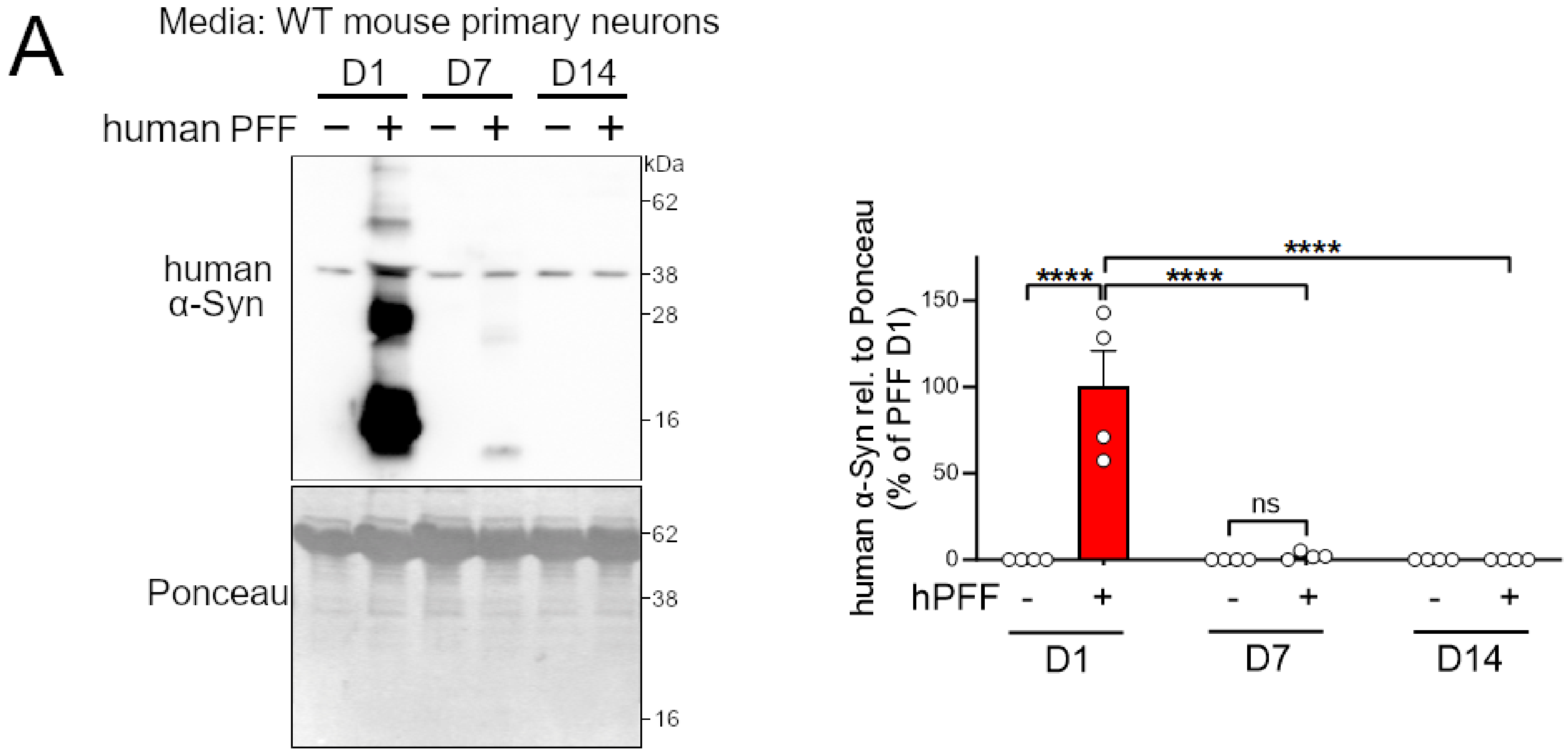
A
Media: WT mouse primary neurons
D1
D7
D14
human PFF
−
+
−
+
−
+
kDa
62
38
28
16
human α-Syn
Ponceau
62
38
16
human α-Syn rel. to Ponceau (% of PFF D1)
150
100
50
0
****
****
****
ns
hPFF
-
+
-
+
-
+
D1
D7
D14

Fig S3A

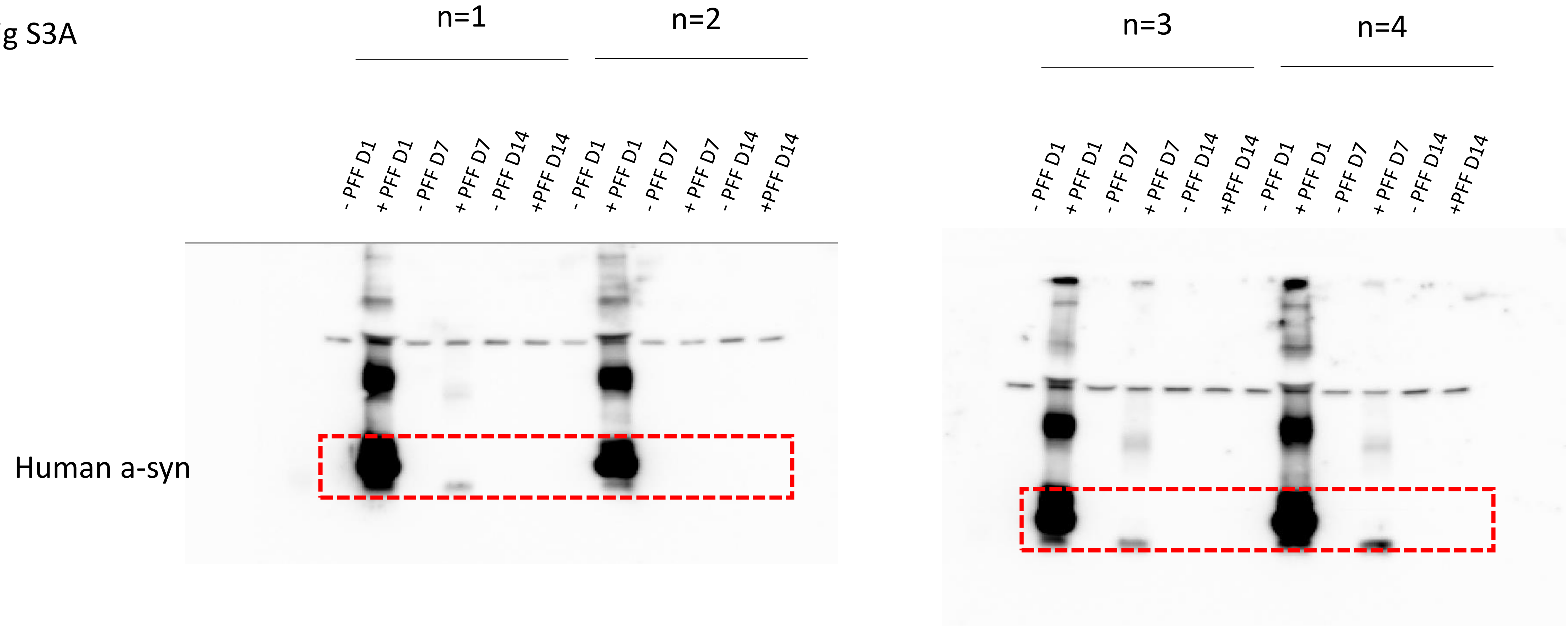

Fig S3A

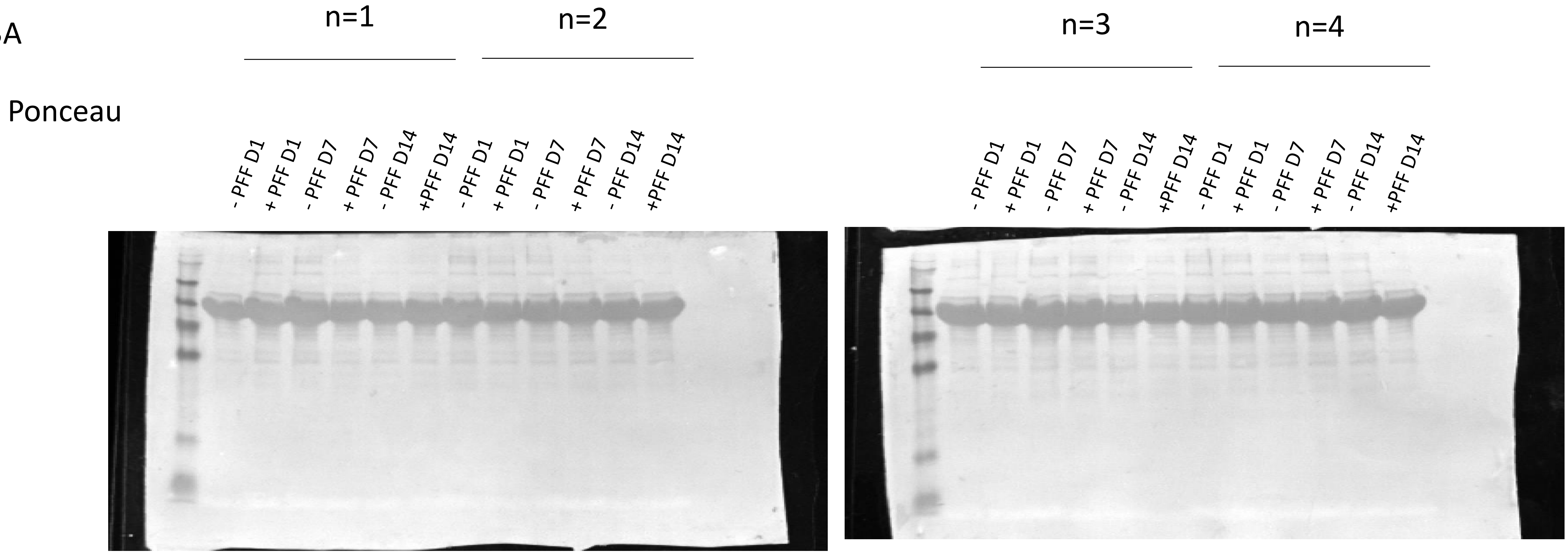

Fig S3B

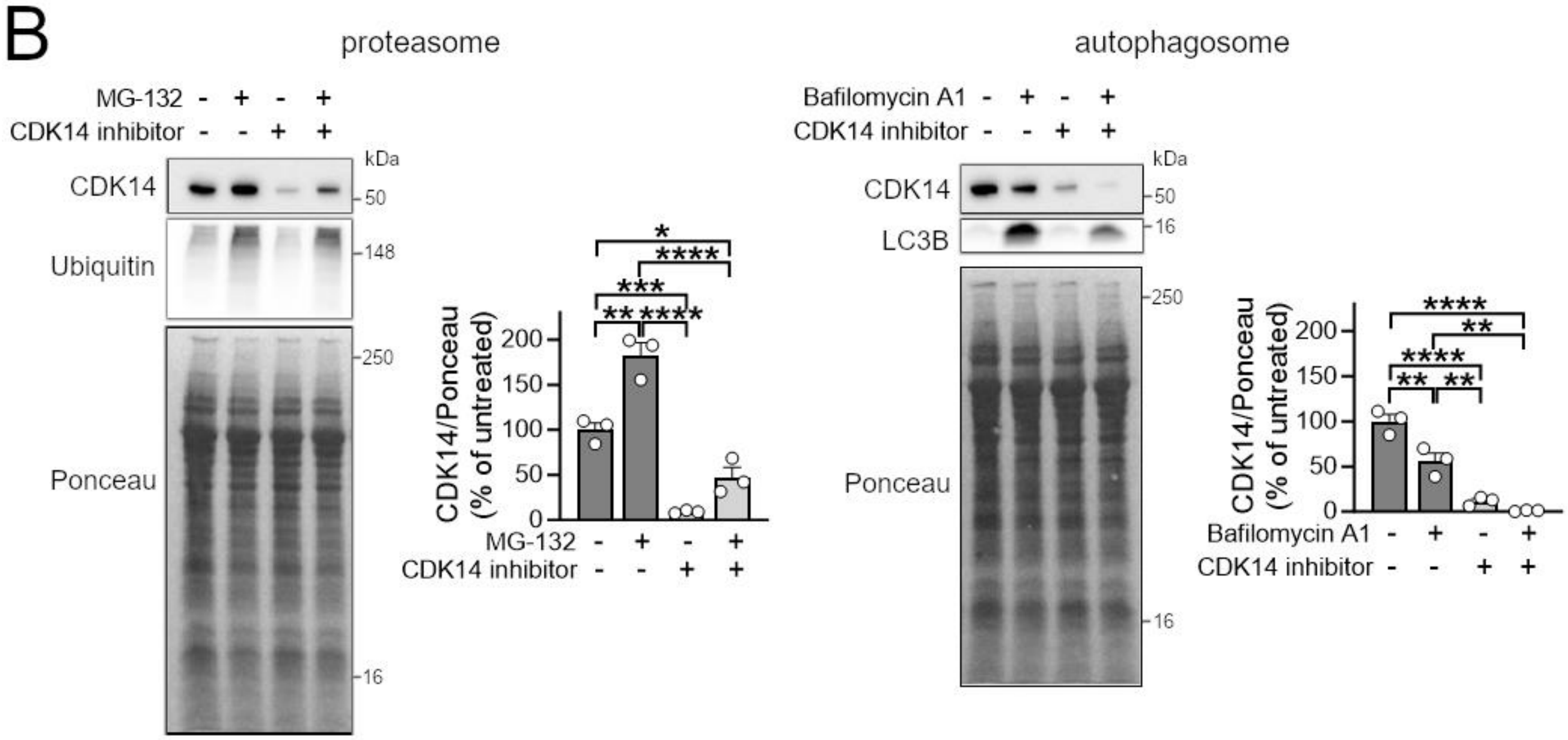
B
proteasome
MG-132 - + - +
CDK14 inhibitor - - + +
kDa
CDK14
50
Ubiquitin
148
Ponceau
250
16
CDK14/Ponceau (% of untreated)
200
150
100
50
0
MG-132 - + - +
CDK14 inhibitor - - + +
autophagosome
Bafilomycin A1 - + - +
CDK14 inhibitor - - + +
kDa
CDK14
50
LC3B
16
Ponceau
250
16
CDK14/Ponceau (% of untreated)
200
150
100
50
0
Bafilomycin A1 - + - +
CDK14 inhibitor - - + +

Fig S3B

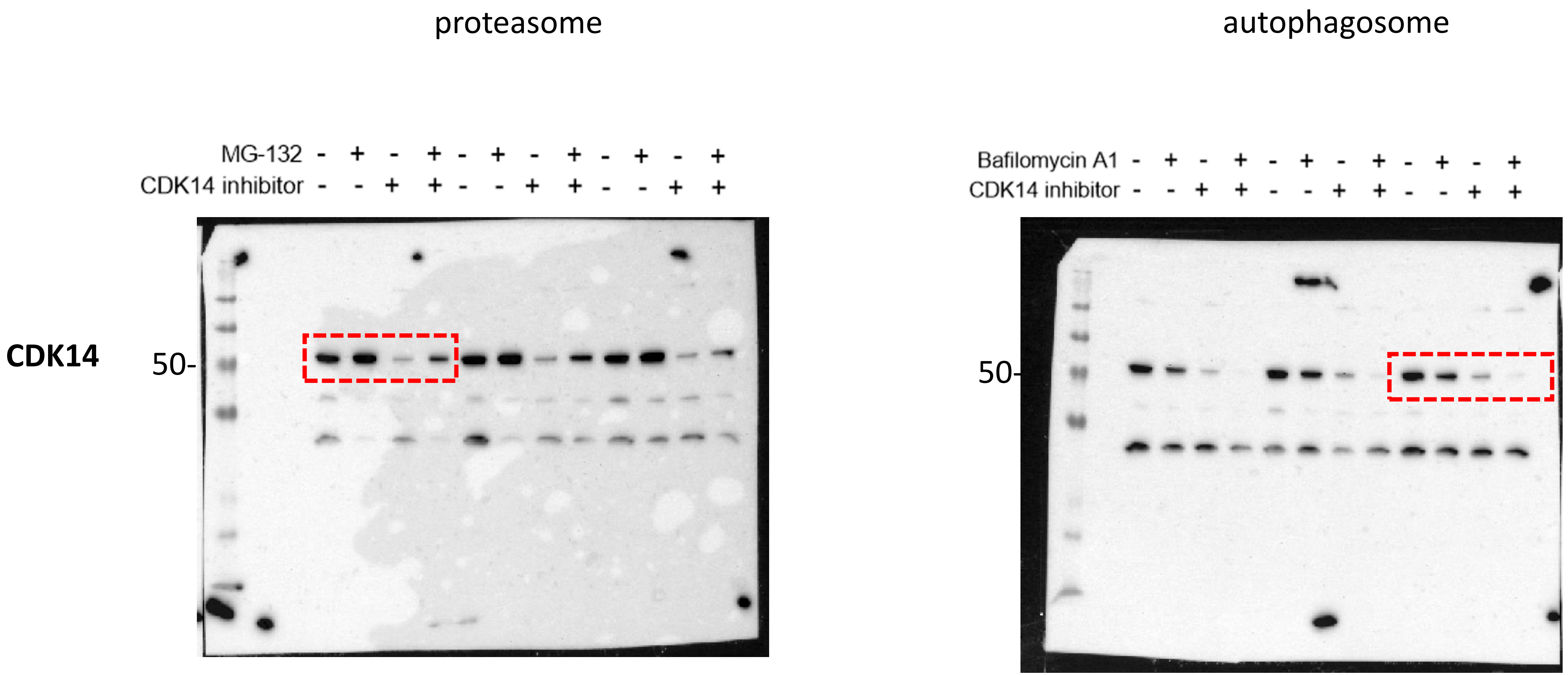

proteasome
autophagosome
MG-132 - + - + - + - + - + - +
CDK14 inhibitor - - + + - - + + - - + +
Bafilomycin A1 - + - + - + - + - + - +
CDK14 inhibitor - - + + - - + + - - + +
CDK14
50-
50-

Fig S3B

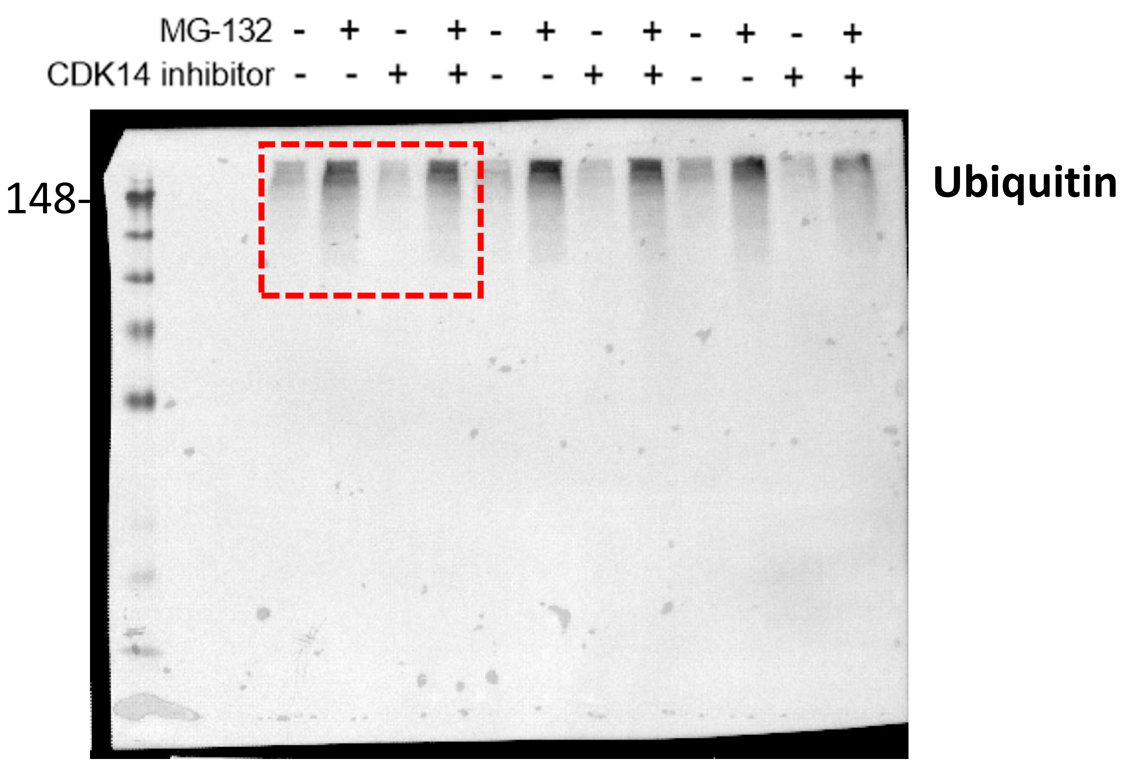
proteasome
MG-132 - + - + - + - + - + - +
CDK14 inhibitor - - + + - - + + - - + +
148-
Ubiquitin

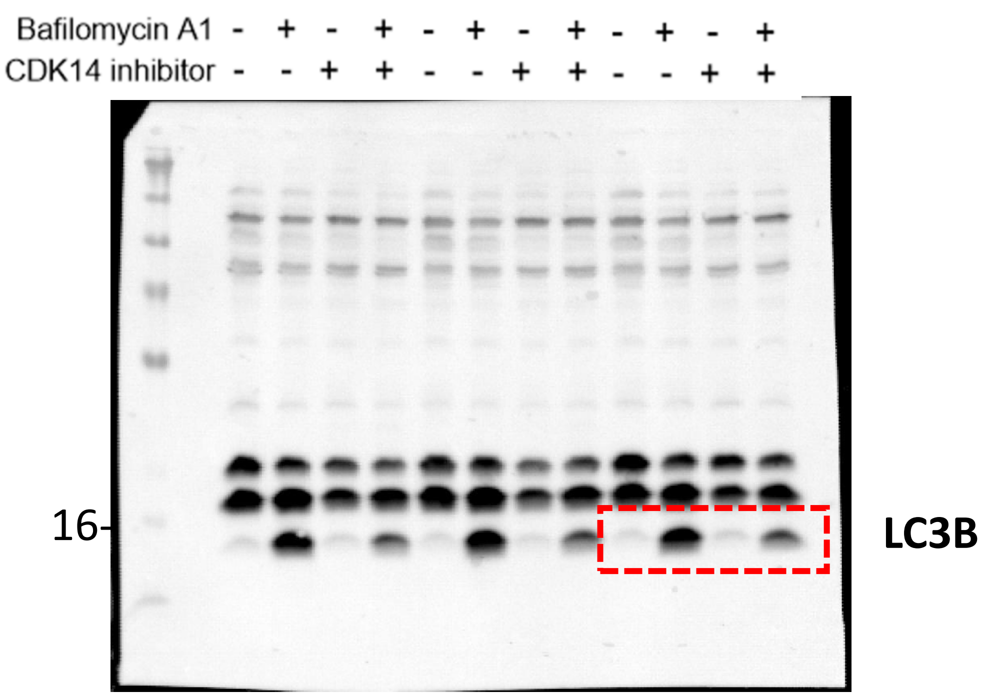
autophagosome
Bafilomycin A1 - + - + - + - + - + - +
CDK14 inhibitor - - + + - - + + - - + +
16-
LC3B

Fig S3B

Ponceau

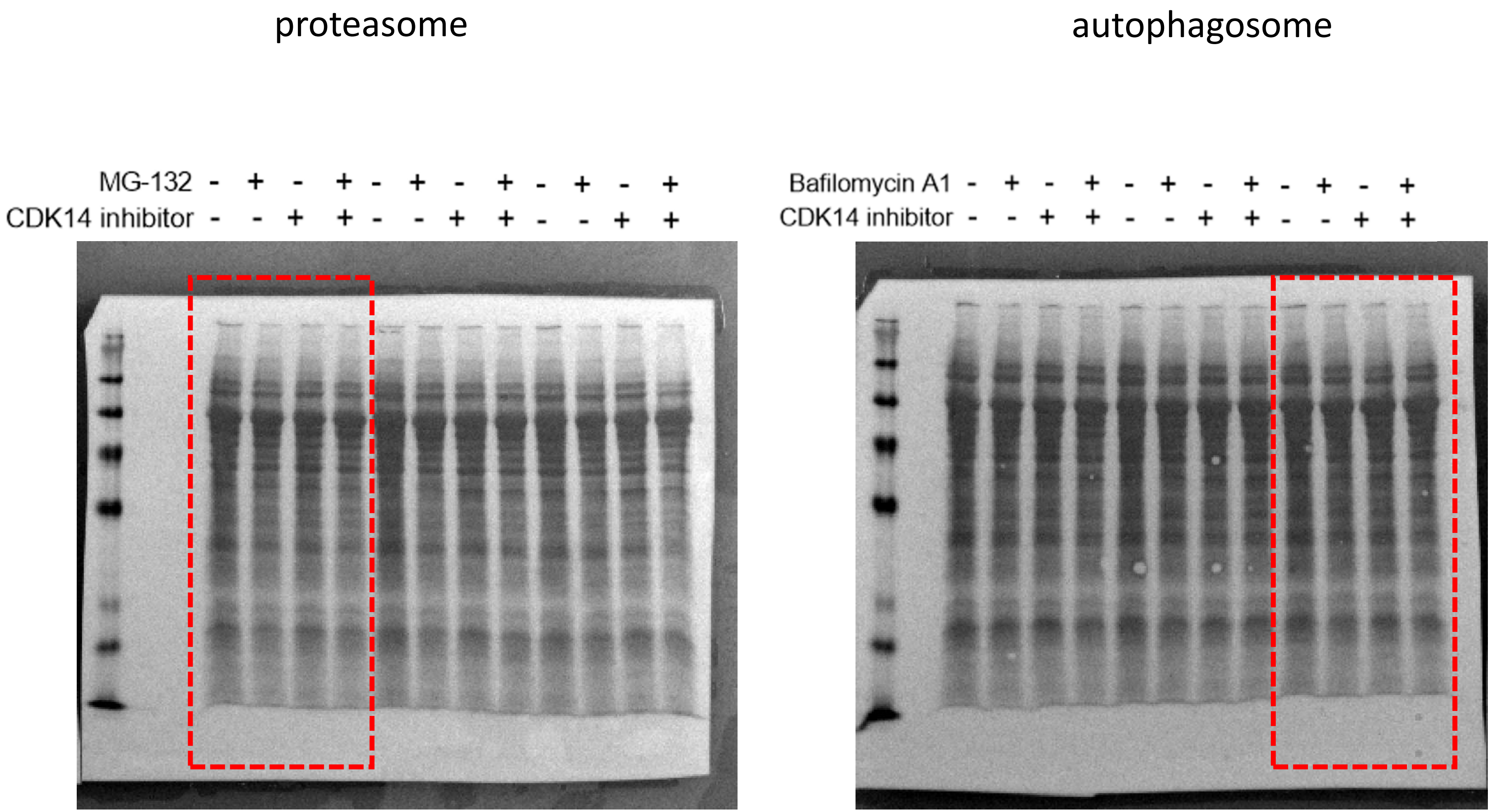

Supplement: Supplementary file 2 — Uncropped western blots [file 41419_2024_6534_MOESM2_ESM.pdf]
